# Conditional genetic screen in *Physcomitrella patens* reveals a novel microtubule depolymerizing-end-tracking protein

**DOI:** 10.1371/journal.pgen.1007221

**Published:** 2018-05-10

**Authors:** Xinxin Ding, Leah M. Pervere, Carl Bascom, Jeffrey P. Bibeau, Sakshi Khurana, Allison M. Butt, Robert G. Orr, Patrick J. Flaherty, Magdalena Bezanilla, Luis Vidali

**Affiliations:** 1 Department of Biology and Biotechnology, Worcester Polytechnic Institute, Worcester, MA; 2 Bioinformatics and Computational Biology Program, Worcester Polytechnic Institute, Worcester, MA; 3 Plant Biology Graduate Program, University of Massachusetts, Amherst, MA; 4 Department of Biological Sciences, Dartmouth College, Hanover, NH; 5 Department of Mathematics and Statistics, University of Massachusetts, Amherst, MA; 6 Department of Biomedical Engineering, Worcester Polytechnic Institute, Worcester, MA; University of California, Riverside, UNITED STATES

## Abstract

Our ability to identify genes that participate in cell growth and division is limited because their loss often leads to lethality. A solution to this is to isolate conditional mutants where the phenotype is visible under restrictive conditions. Here, we capitalize on the haploid growth-phase of the moss *Physcomitrella patens* to identify conditional loss-of-growth (CLoG) mutants with impaired growth at high temperature. We used whole-genome sequencing of pooled segregants to pinpoint the lesion of one of these mutants (*clog1*) and validated the identified mutation by rescuing the conditional phenotype by homologous recombination. We found that *CLoG1* is a novel and ancient gene conserved in plants. At the restrictive temperature, *clog1* plants have smaller cells but can complete cell division, indicating an important role of *CLoG1* in cell growth, but not an essential role in cell division. Fluorescent protein fusions of CLoG1 indicate it is localized to microtubules with a bias towards depolymerizing microtubule ends. Silencing *CLoG1* decreases microtubule dynamics, suggesting that CLoG1 plays a critical role in regulating microtubule dynamics. By discovering a novel gene critical for plant growth, our work demonstrates that *P*. *patens* is an excellent genetic system to study genes with a fundamental role in plant cell growth.

## Author summary

Genes important for cell growth are difficult to identify because their disruption often results in the death of the organism. A solution to this problem is to isolate temperature-sensitive mutants where growth is blocked only at high temperatures. Here, we used the moss *Physcomitrella patens*, a simple model plant, to isolate temperature-sensitive mutants with reduced growth. We used whole-genome sequencing to identify the gene disrupted in one of these mutants (*clog1*). We found that *CLoG1* is a previously uncharacterized gene present in algae and plants. Localization studies of CLoG1 protein in living cells showed CLoG1 concentrates on microtubules and tracks depolymerizing ends. Loss-of-function analysis suggests a possible role in controlling microtubule dynamics. Our approach establishes the moss *P*. *patens* as a valuable model-organism to investigate genes important for cell growth in plants.

## Introduction

Early adopters of *P*. *patens* as a genetic model plant identified its haploid genetics as a valuable attribute for genetic analysis. Mutants displaying a variety of defects, including metabolic and hormonal deficiencies as well as morphological and physiological alterations, were easily isolated using simple mutagenesis [1–3]. Despite the success in isolating mutants, identification of the causal mutations was not readily achieved until recently with the advance of whole-genome sequencing and the availability of polymorphic strains [4]. Similar to other systems, mapping can be rapidly achieved by pooling the mutant DNA from segregants resulting from crosses between polymorphic strains and sequencing the segregants’ genomes, providing an immediate map to identify the location of a mutation with high accuracy [4–7].

Although the predominant haploid growth phase of *P*. *patens* is valuable for genetic screening, identifying mutations in essential genes, including genes important for cell growth and division, can be complicated. To overcome these limitations it is possible to isolate conditional mutants, which has been an effective approach to study genes that are essential for growth and viability in a number of organisms [8–13]. Temperature-sensitive (TS) conditional mutants display phenotypic defects under restrictive temperatures. TS mutants have not been widely used in plants, but some important studies–show their great potential for investigating plant genes important for growth [14–17] and microtubule dynamics in *Arabidopsis thaliana* [18–20].

Among many essential cellular structures, the microtubule cytoskeleton plays a prominent role in organizing plant cell growth and division. Subcellular arrays, such as the mitotic spindle and the phragmoplast, are critical for proper chromosome segregation and cytokinesis, respectively [21, 22]; while the cortical microtubule array is involved in cellulose deposition and the delivery of other cell wall components [23, 24]. For the microtubule cytoskeleton to function, it is necessary that the interaction of motors, bundling proteins, severing proteins, and end binding proteins are regulated in a dynamic fashion [25–27]. Many of these microtubule-associated proteins are conserved in plants and shown to have similar function to their animal and fungal homologues [28]. Nevertheless, it has also been shown that the plant microtubule cytoskeleton has unique forms of regulation and associated proteins not found in cells of other organisms [29]. Due to its complexity, our understanding of the composition and regulation of the plant microtubule cytoskeleton still requires additional investigation.

Here, to identify genes important for plant cell growth and division, we aimed to isolate TS mutants from *P*. *patens* and identify the causal mutation using pooled segregant analysis and next-generation whole-genome sequencing. We used ultra-violet (UV) light-induced mutagenesis and screened mutants with impaired growth by separating them by size. We isolated several mutant plants that grow normally at room temperature (20–25°C) but had reduced growth at 32°C. We selected one mutant with reduced cellular growth for detailed characterization and identified the mutation responsible for the TS phenotype. Highlighting the potential importance of our approach, the gene we identified was previously uncharacterized, but is conserved in slime molds, algae, and plants. Interestingly, this novel protein localizes to the microtubule cytoskeleton in *P*. *patens*, and tracks depolymerizing microtubules ends. RNA-based loss-of-function analysis suggests a possible role in the regulation of microtubule dynamics. Our TS mutant screen allowed us to discover a novel protein conserved throughout evolution and important for plant cell growth.

## Results

### Isolation and screening of temperature-sensitive mutants

**Fig 1:**
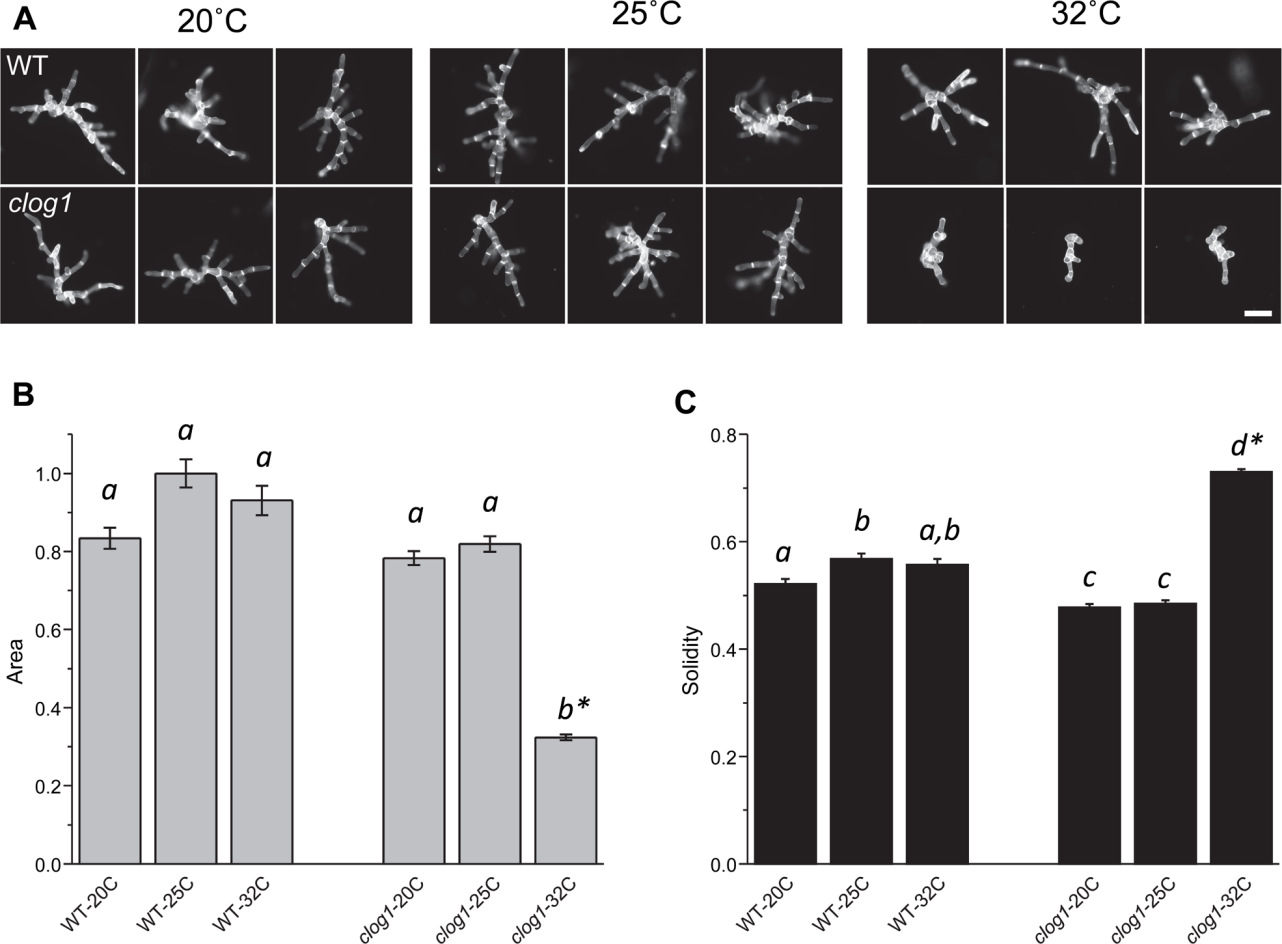
Mutant *clog1* plants show evidence of growth defects at 32°C. **(A)** Three representative micrographs of indicated moss plants that were grown at 20°C, 25°C, and 32°C. In growth assays, the plants were photographed on day 3 after being passed onto growth medium and grown at the indicated temperatures. Bar = 100 μm. **(B)** and **(C)** Plots show normalized area and solidities of mutant and control at 20°C, 25°C, and 32°C on day 3 after passing onto growth medium. Standard error of the mean is shown. Letters on top of the bar show groups that cannot be distinguished statistically by one-way ANOVA-Tukey (P <0.05). Asterisk indicate a P <0.01 against all other groups. Plant sample sizes are as follows: WT-20C:279, WT-25C:375, WT-32:256, *clog1*-20C:457, *clog1*-25C:557, *clog1*-32C:522.

To isolate temperature-sensitive (TS) mutants, we first identified a temperature span that allows wild type *P*. *patens* plants to grow to a similar extent and have similar morphology. By comparing the plant area and morphology, we found wild type plants grow similarly between 20°C and 32°C (**Fig 1**). To identify TS mutants, we selected our standard culturing temperature of 25°C as the permissive temperature, and the maximum temperature of 32°C as the restrictive temperature. To isolate mutant plants, we irradiated protoplasts with UV light to induce mutations, optimizing the amount of irradiation to obtain approximately 90–95% killing frequency [30]. Protoplasts regenerated their cell walls for four days at 25°C and then were cultured for one week at 32°C to induce potential TS defects. Following this regeneration and culture period, we isolated plants smaller than 200 μm in diameter by filtering all the regenerated plants through a sieve. We grew these small plants at 32°C for an additional week to discard the background non-TS plants that grow under these conditions. We manually selected small plants that fail to grow at 32°C and distinguished TS plants from non-TS mutant plants by their ability to resume growth upon transfer to 25°C (**S1 Fig** and Materials and Methods). To confirm temperature-sensitivity, we compared the growth of each putative TS mutant against wild type plants by expanding the isolated mutants onto two agar plates and culturing the plants at 25ºC and 32ºC. From three initial screens, where approximately 5,000 mutant plants were screened in each, we obtained an average yield of six TS mutant plants per screen. We named these genes *CLoG* for Conditional Loss of Growth and selected the mutant plant *clog1*, which expresses a strong TS phenotype, for additional analysis.

### Morphological characterization of the TS mutant *clog1*

To obtain quantitative growth and morphological information, we performed growth assays on the *clog1* mutant [31]. Protoplasts were regenerated for four days, transferred to growth medium, and assayed for growth at 20°C, 25°C, and 32°C. Three days after transfer to growth medium, we stained the cell walls of the regenerating plants with calcoflour, and imaged them with epifluorescence microscopy [31]. The mutant and wild type plants grew similarly at 20°C and 25°C and only *clog1* exhibited an inhibition of plant growth at 32°C (**Fig 1**). We measured plant area and solidity (area/convex hull area), using total area to assess growth rate and solidity to assess the extent of polarization and branching of protonemata filaments [31]. These data show that *clog1* is a TS mutant for growth, demonstrating that by using a simple sieving and temperature selection screening system (**S1 Fig**) we can isolate TS mutant plants of *P*. *patens* with altered growth at the restrictive temperature.

**Fig 2:**
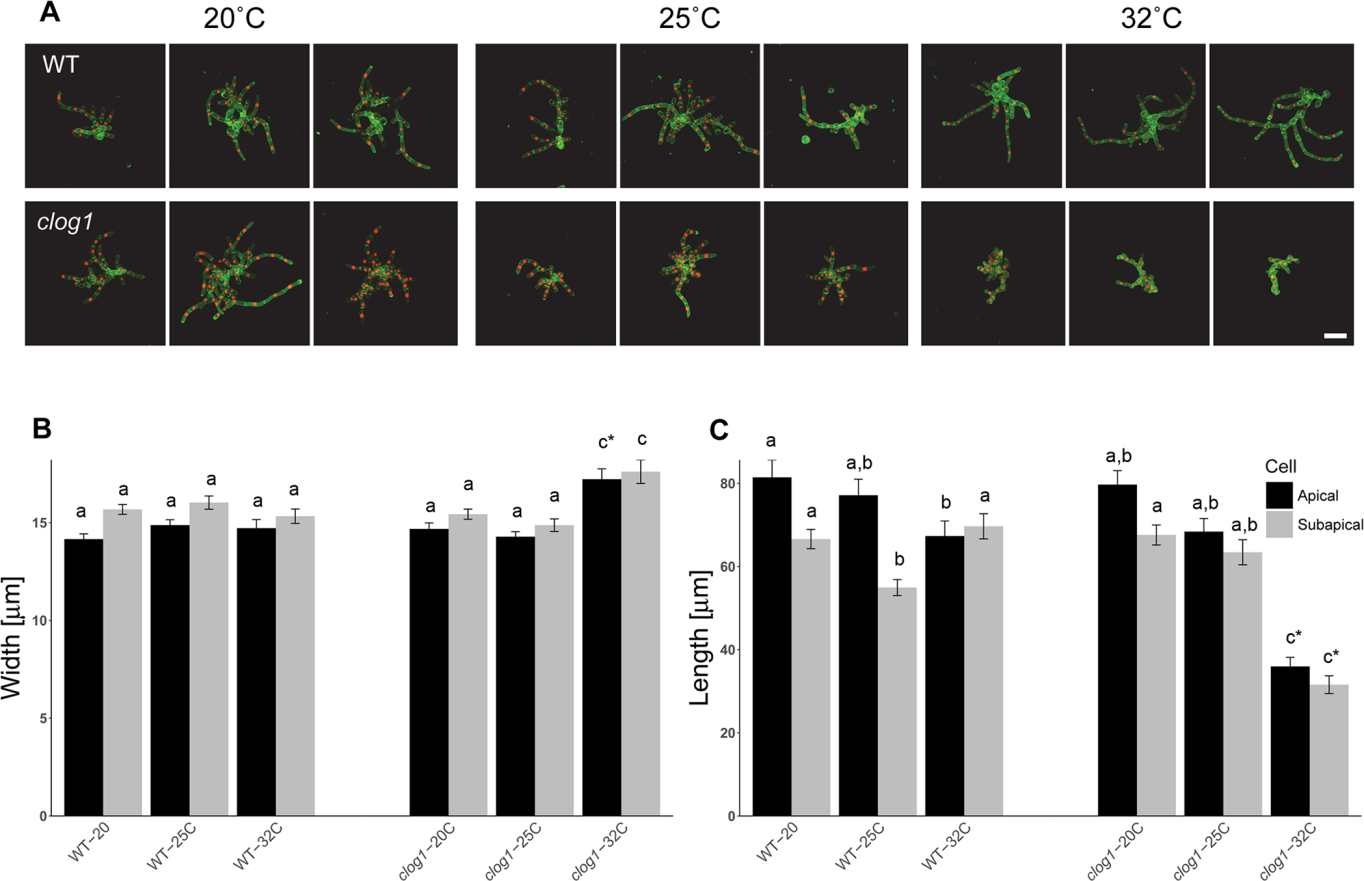
Mutant *clog1* plants show reduced cell growth, but no multinucleated cells at 32°C. **(A)** Three representative micrographs of indicated moss plants that were grown at 20°C, 25°C, and 32°C. The plants were imaged on day 3 after being passed onto growth medium and grown at the indicated temperatures. Bar = 100 μm. **(B)** and **(C)** Plots show width and length of the apical and subapical cells from the longest filament in the mutant and control plants at 20°C, 25°C, and 32°C on day 3 after passing onto growth medium. Standard error of the mean is shown. Letters on top of the bar show groups that cannot be distinguished statistically by one-way ANOVA-Tukey (P <0.05). Asterisk indicate a P <0.01 against all other groups, sample sizes are 40 cells for all conditions.

To further characterize the cellular basis of the reduced plant growth, we measured cell size and investigated possible cell division defects. We analyzed plants at the same stage and temperature as indicated above using three-dimensional reconstruction (see Materials and Methods section). To evaluate cell size, we stained the cell walls, and to evaluate the presence of multinucleated cells, we generated *clog1* cell lines expressing a GFP-GUS fusion with a nuclear localization signal [32]. The apical and sub-apical cells of the longest filaments showed a significant reduction in length, which was accompanied by an increase in width (**Fig 2A–2C**). The change in width was not compensatory, because the final volume of the *clog1* cells at the restrictive temperature was smaller than in control cells. These results suggest a role for CLoG1 protein in cell polarization and growth. With regard to cell division, we did not observe multinucleated cells in *clog1* plants grown at the restrictive temperature (**Fig 2A**), indicating that CLoG1 is not essential for completing cell division.

### Mapping of *clog1* by outcrossing and genome sequencing of segregants

A critical limitation that has hindered the establishment of *P*. *patens* as a forward genetic system is the inability to map and subsequently identify a mutated allele. Here we chose genome sequencing of pooled segregants as the strategy to identify the causal mutation for the *clog*1 TS mutant [6, 7]. By only selecting segregants that display the TS phenotype, the causal mutation remains with the segregants while other parts of the genome undergo random chromosomal crossover and recombination during meiosis. Therefore, genomic recombination rates should decrease in frequency for regions closer to the causal mutation. We generated a mapping population by outcrossing *clog1* plants (Gransden strain) to a polymorphic Villersexel strain [33], which expresses soluble mCherry (Vx::mCherry). We identified the crossed sporophytes by mCherry fluorescence of the capsule on a non-fluorescent TS mutant gametophyte [33].

Mapping using whole-genome sequencing is most successful with a large enough mapping population. To determine the appropriate size of our mapping population, we designed a Monte Carlo simulation exploring the relationship between the size of the mapping population (number of segregants) and the size of the mapping interval—the region potentially containing the causal mutation (for details see Materials and Methods section). The simulation was based on an approximately 450Mbp genome consisting of 27 chromosomes (*Physcomitrella patens v3*.*0 early release*) and a recombination frequency per chromosome of zero, one, or two[34]. As seen in **S2 Fig**, the magnitude of the decrease in median mapping interval size became smaller when the mapping population size was increased from 20 to 30 and even smaller when the population size increased from 40 to 90. Based on the simulation results, and given good sequencing quality with enough depth (10x coverage), the causal mutation should reliably be mapped onto one chromosome within 1–3 Mbp. This conclusion is based on a mapping population of 24 F1 *clog1* segregants and an approximately 450 Mbp genome consisting of 27 chromosomes.

To identify and pool the segregants of *clog1*, we screened outcrossed plants at 25°C and 32°C for loss of growth at 32°C. After screening 120 F1 segregants, 24 were selected that exhibited a robust TS phenotype similar to that of *clog1* at 32°C (Materials and Methods). It is important to note that the precise segregation ratio was difficult to estimate because, to reduce any possible background, we discarded any plants that could not be clearly assigned a TS phenotype. To identify the approximate location of the *clog1* mutation, we extracted, pooled, and sequenced genomic DNA from the 24 F1 progeny of the *clog1*-Vx::mCherry cross (Materials and Methods). We identified single nucleotide polymorphisms (SNPs) as markers that defined differences between the Villersexel and Gransden genomes to measure genomic recombination in pooled segregants. The reference (Gransden) allele frequency at marker positions was used to map the *clog1* mutation. We expected that the reference allele frequency would be highest in regions close to the causal mutation and would be approximately 0.5 in the rest of the genome assuming that random recombination occurs. We also calculated marker densities (1 marker every 200 /bp) and average read depth (8X) to assist in assessment of reference allele frequencies at different chromosomal positions (Materials and Methods).

We mapped the suspected causal mutation and the gene where it is located after aligning the reads to *P*. *patens* genome assembly V1.2 [35], as this was the only assembly for which the genome annotation file was publicly available. We selected a total of 2,292,625 SNP markers by comparing the genome sequences of the *P*. *patens* Gransden and Villersexel strains, at an average of one marker per 207 bp. At the marker positions, we detected 1,722,037 SNPs (75.1% of all markers) in the pooled segregants’ genome sequence. We used a MATLAB routine to visualize reference allele frequencies, marker densities, and average read depth across all 27 chromosomes (Materials and Methods). We conducted these calculations for every non-overlapped 40 Kbp window.

**Fig 3:**
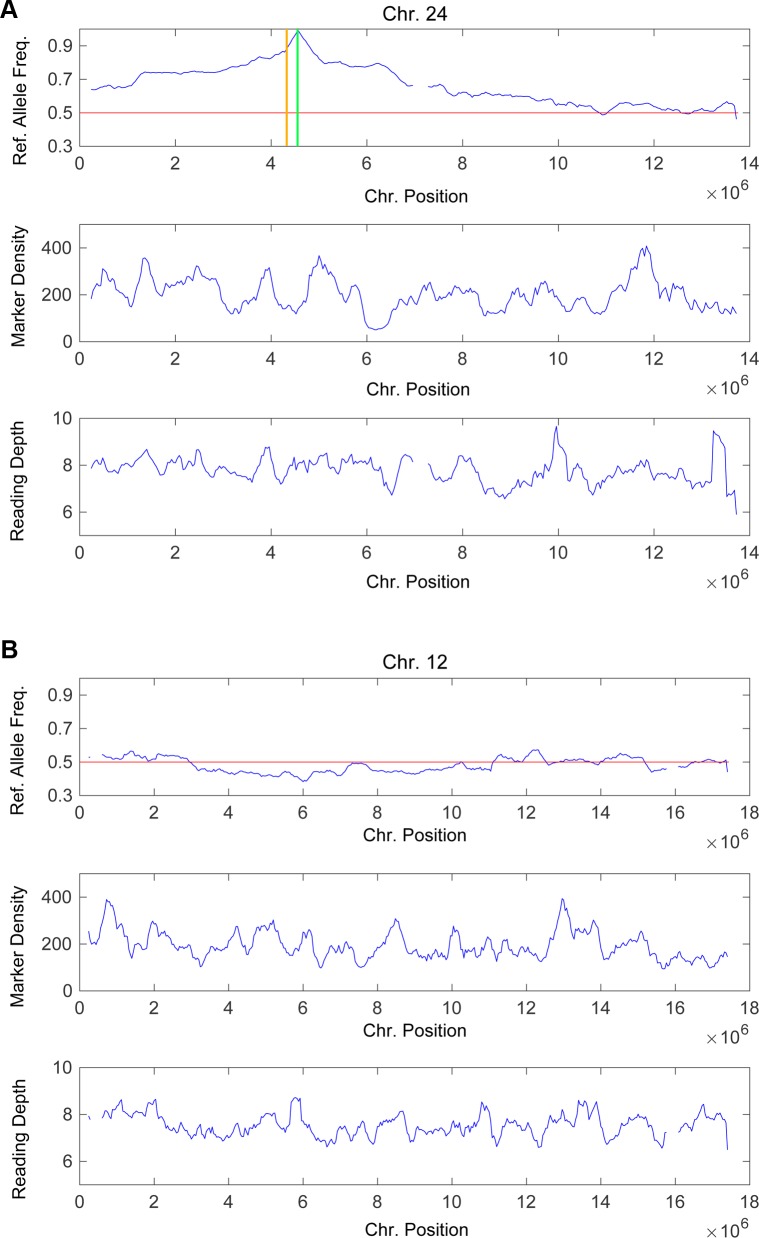
The causal mutation of *clog1*’s temperature-sensitivity is located on chromosome 24. The causal mutation is located by calculating the reference (Gransden) allele frequencies, marker (Villersexel SNP) densities, and average read depth using sliding windows of 40,000 bp for all 27 chromosomes. The curves were generated using a simple moving average model with a window size of 7. **(A)** Reference allele frequencies, marker densities, and average reading depth per nucleotide locus of chromosome 24. The green and orange lines in the top plot of panel A indicate the peak (around 4,563,000 bp) of the reference allele frequency and the predicted causal mutation (4,325,703 bp) respectively. **(B)** Reference allele frequencies, marker densities, and average reading depth per nucleotide locus of chromosome 12. The red line in the top plot of each panel indicates the reference allele frequency of 0.5.

We found that chromosome 24 was the only chromosome whose reference allele frequency reached one at a particular position (**Fig 3**), approximately 4.6 Mbp into chromosome 24 (the green line in **Fig 3A**). On both sides of this peak, the reference allele frequencies gradually increase from 0.5 on the right side and 0.6 on the left side to 1.0. We did not observe a similar pattern on any of the other chromosomes, where the reference allele frequencies mostly fluctuated around 0.5 (**Fig 3B** shows chromosome 12 as a representative). Additionally, the marker densities and average read depth of all chromosomes fluctuated around 200 markers per 40 Kbp window (one marker per 200 bps) and 8X coverage respectively (**Fig 3**). This is very close to the average marker density of one marker per 207 bp and the genome coverage of 9.2X determined from the alignment of the pooled segregants’ genome, indicating that markers were generally evenly distributed across the 27 chromosomes and that most regions of every chromosome were supported by eight reads. Taken together these data identified the mapping interval for the causal mutation of *clog1* as a 1 Mbp segment (located at 4.1–5.1 Mbp) centered at the peak of reference allele frequency (4.6 Mbp) on chromosome 24.

With such a large mapping interval (30–40 genes) it is not possible to identify a single gene. Instead, we reasoned that the causal mutation is most likely a non-synonymous SNP in the open reading frame of a gene. There are two main reasons for this: first, point mutations are one of the most common signature mutations of UV mutagenesis [36], and second, the causal mutation is likely to cause an amino acid change (missense mutation) in a functional protein because the protein conformational change and resulting growth defect only take place at high temperature. Therefore, we filtered for non-marker and non-synonymous SNPs within the mapping interval on chromosome 24, which was covered by scaffolds 73, 274, and 387 of the V1.2 genome assembly (Materials and Methods). We found one mutation in scaffold 387 that fulfills these requirements. The mutation is located at position 4,325,703 of chromosome 24 (*Physcomitrella patens v3*.*0 early release*) (orange line in **Fig 3A**), which is approximately 270 Kbp from the peak of the reference allele frequency. This mutation mapped to gene Pp1s387_7V6 [Pp3c24_6470, Genbank MG754010] and was predicted to cause an amino acid change at position 874 from a leucine to a phenylalanine; the length of the ORF is 3,822 bp (1,274 amino acids). The function of Pp3c24_6470 is currently unknown and there is only one copy of this gene in *P*. *patens* [35]. Additionally, no conserved domain of known function has been identified in the protein encoded by this gene. We identified homologous proteins in amoeboid protists (*Dctyostelium fasciculatum*, *Dyctostelium discoideum*, and *Polysphondylium pallidum*), green algae, and land plants. The Panther Classification System classifies CLoG1 in the unnamed gene family PTHR34958. Interestingly, with only a few exceptions, the gene is present as a single copy in most of the species analyzed (**S1 Table**).

Phylogenetic analysis of the amino acid sequences groups the proteins homologous to CLoG1 with the expected clades of protists, bryophyte, monocots, etc. (**S3 Fig**). Amino acid composition shows an abundance of leucine residues (~12%), secondary structure prediction shows the propensity for the presence of alpha-helices and no coiled-coil formation (**S4 Fig**). To evaluate the most conserved regions of the protein we aligned the amino acid sequences of two amoeboid protists, two green algae, and two land plants. Interestingly, the alga sequences are significantly longer that their plant or protist counterparts. We selected six highly conserved regions containing 20% or more identical residues between all six species. These regions are indicated on the *P*. *patens* sequence in **S4 Fig** and the alignments shown in **S5 Fig**. Besides the presence of abundant leucine residues in all these regions, no other obvious sequence motifs were observed. The longest conserved regions are located at the N and C termini of the molecule.

### *CLoG1* locus verification by genetic rescue

**Fig 4:**
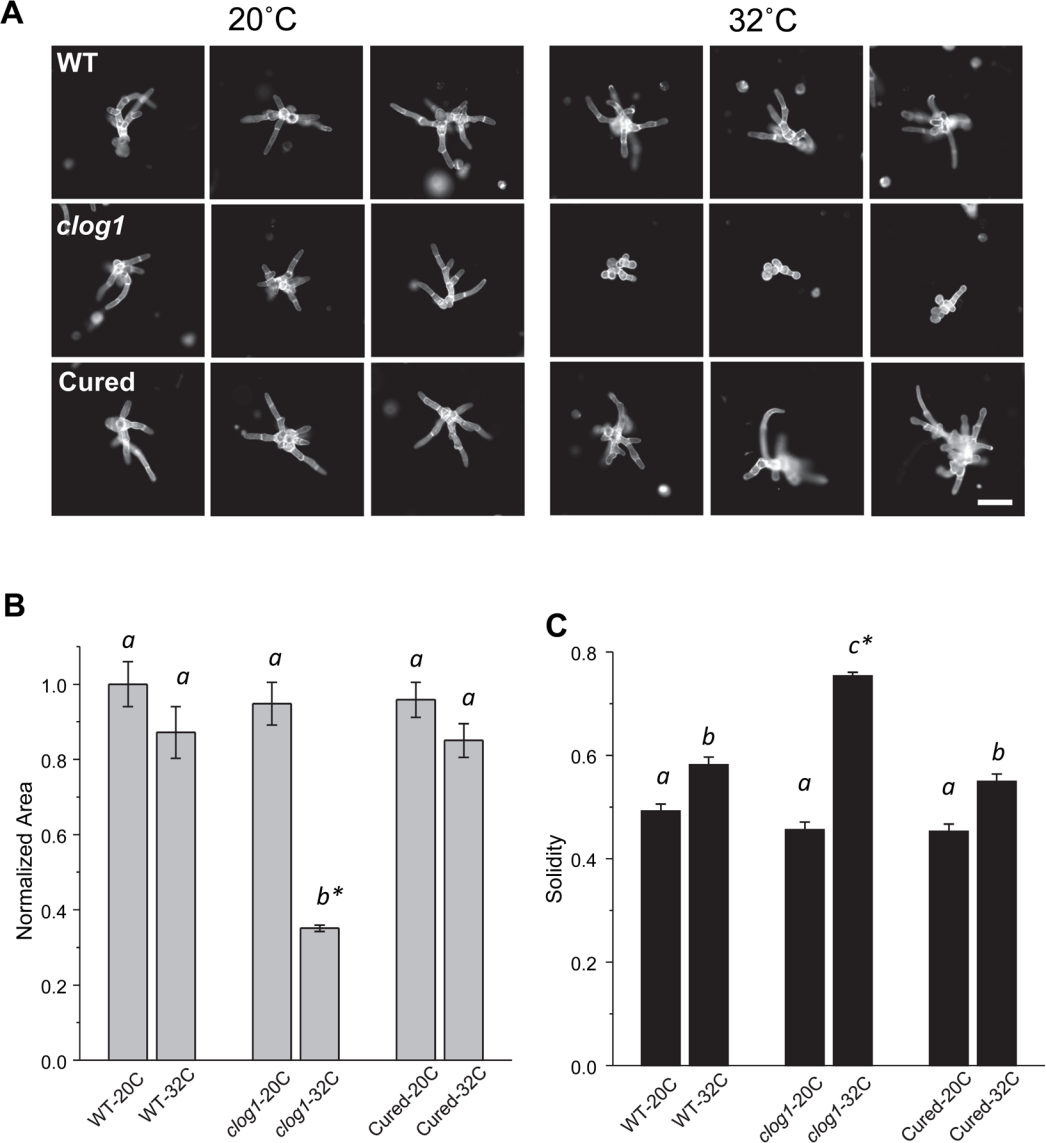
Rescue of *clog1* temperature-sensitive phenotype using homologous recombination. **(A)** Representative images of control, *clog1* mutant plants, and rescued plants at the permissive and restrictive temperatures. The mutation in the *clog1* conditional allele was repaired using a PCR product from the wild type plant. Scale Bar = 100 μm. **(B)** and **(C)** Plots show normalized area and solidities of control, mutant and rescue plants at 20°C and 32°C on day 3 after passing onto growth medium. Standard error of the mean is shown. Letters on top of the bar show groups that cannot be distinguished statistically by one-way ANOVA-Tukey (P <0.05). Asterisk indicate a P <0.01 against all other groups. Plant sample sizes are as follows: WT-20C:198, WT-32C:158, *clog1*-20C:141, *clog1*-32C:337, Cured-20C:186, Cured-32C:178.

To confirm that the mutation identified was responsible for the TS *clog1* phenotype, we used homologous recombination to replace a 2kb genomic region flanking the putative *clog1* point mutation with a wild type genomic fragment. We used PCR to amplify the 2kb fragment and transformed the PCR product into *clog1* mutant plants. We co-transformed a circular plasmid with hygromycin resistance to help select for transformed plants. To confirm the replacement, via homologous recombination, of the mutant allele with the wild type allele, we sequenced the amplified region in twenty transformed plants and identified one plant with the wild type sequence. We verified that this plant was of the mutant’s genetic background—and not a result of contamination with wild type DNA, by sequencing a second mutant locus in chromosome 24 which exhibited the mutant’s genetic background. Using a growth assay, we confirmed genetic reversion of the conditional loss-of-growth phenotype (**Fig 4**). Together, these results strongly support that we identified the causal mutation responsible for *clog1*.

### CLoG1 localizes to the microtubules and accumulates at depolymerizing ends

**Fig 5:**
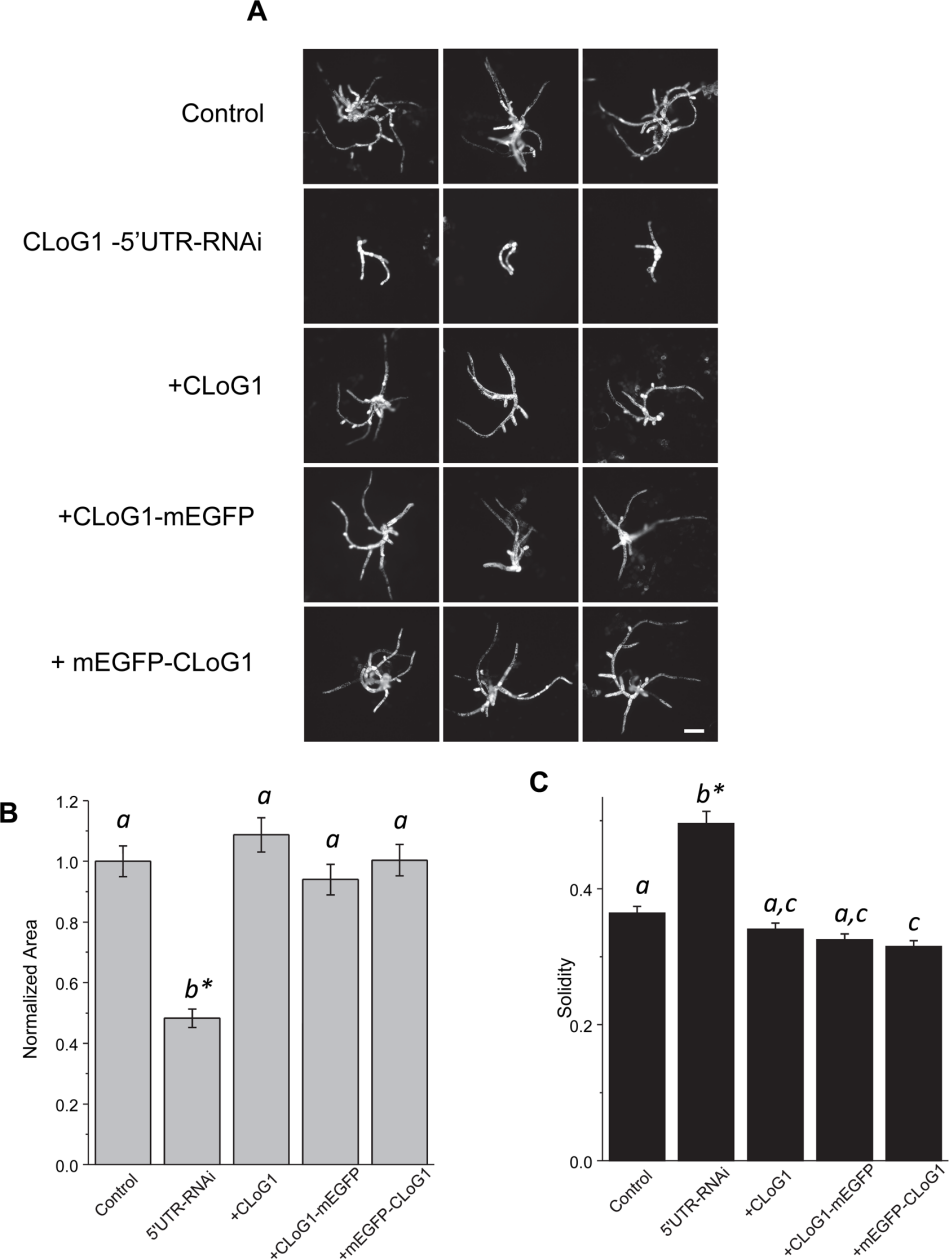
Fluorescent protein fusions of CLoG1 are functional. Transient RNAi-based silencing of *CLoG1* and transient complementation using CLoG1 mEGFP fusions were used to test for protein fusion functionality. All experiments were done at room temperature. **(A)** Representative images of control, control-RNAi plants, plants transformed with the CLoG1-RNAi construct, and plants complemented with the CLoG1 cDNA and C- and N- terminal fusions. Scale Bar = 100 μm. **(B)** and **(C)** Plots show normalized area and solidities of control-RNAi, CLoG1-RNAi and complemented plants on day 3 after passing onto growth medium. Standard error of the mean is shown. Letters on top of the bar show groups that cannot be distinguished statistically by one-way ANOVA-Tukey (P <0.05). Asterisk indicate a P <0.01 against all other groups. Plant sample sizes are as follows: control-RNAi:88, 5´UTR-RNAi:90, +CLoG1:98, +CLoG1-mEGFP:102, +mEGFP-CLoG1:88.

To gain insight into the intracellular function of *CLoG1*, we generated CLoG1 proteins fused to monomeric enhanced green fluorescent protein (mEGFP) and determined their subcellular localization. To evaluate whether the fluorescent protein fusions are functional, we took advantage of a well-established transient RNAi and complementation assay [37, 38]. We generated an RNAi construct that targets the 5´UTR of *CLoG1*. Importantly, we found that plants transformed with the CLoG1-UTR RNAi construct show, at room temperature, a similar loss-of-growth phenotype to *clog1* mutants grown at the restrictive temperature (**Fig 5**). By co-transforming CLoG1-UTR RNAi with a construct that constitutively expresses the *CLoG1* open reading frame, we observed complete rescue of the RNAi phenotype (**Fig 5**). We also observed that either C-terminal and N-terminal fusions of CLoG1 to mEGFP fully complemented the CLoG1-RNAi plants, demonstrating that both fusion proteins are functional (**Fig 5**). Finally, *clog1* plants expressing a C-terminal fusion of CLoG1 to mEGFP complement the TS phenotype, further confirming that the fusion protein is functional and demonstrating that the *clog1* allele is recessive (**S6 Fig**).

**Fig 6:**
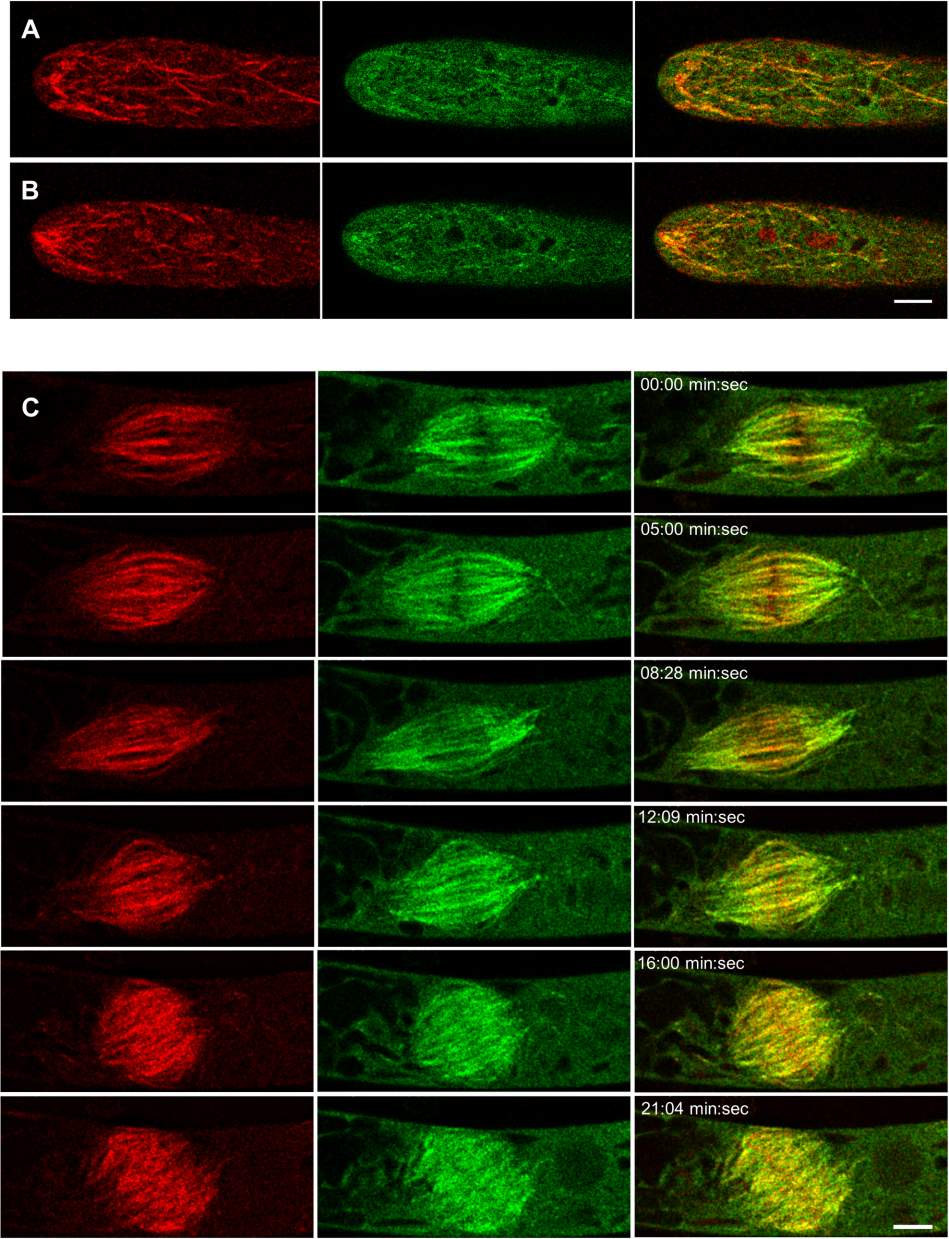
CLoG1 fluorescent protein fusions localize to the microtubule cytoskeleton. **(A)** Laser confocal images of a growing caulonema cell expressing mCherry-tubulin (red) and CLoG1-mEGFP (green), cortical optical section are shown. **(B)** Laser confocal images of a growing caulonema cell expressing mCherry-tubulin (red) and CLoG1-mEGFP (green), medial optical section are shown. **(C)** Representative example of CLoG1-mEGFP and mCherry tubulin during mitosis and early cytokinesis. Note that CLoG1-mEGFP (green) is present in the whole spindle but with a bias toward the spindle poles in relation to mCherry-tubulin (red); this is most obvious during anaphase (8:28 min:sec). Scale bars = 5 μm.

We attempted to generate lines with the endogenous locus tagged with mEGFP, which we have shown is functional. Unfortunately, the resulting fluorescence levels in the properly tagged lines were too low to detect above background using our confocal microscope system. Low levels of CLoG1 protein are consistent with a low level of transcript expression deduced from the transcriptome atlas of *P*. *patens* [39]. Therefore, to observe the intracellular localization of CLoG1-mEGFP, we generated stable lines in the wild type background where CLoG1-mEGFP was driven by a constitutive promoter [37]. For imaging, we selected plants with normal growth. Strikingly, we found that CLoG1 localizes to filamentous structures that resemble the microtubule cytoskeleton [40–42]; we could identify filaments immediately below the plasma membrane, as well as in the cytoplasm (**Fig 6A and 6B**). To identify if CLoG1 localizes with specific sub-structures of the microtubule cytoskeleton, we expressed mCherry-labeled alpha-tubulin, which integrates into dynamic microtubules, in the CLoG1 C-terminal mEGFP fusion line. In growing apical caulonema cells, CLoG1-mEGFP appeared to only localize to sections of microtubules, sometimes forming punctate structures (**Fig 6A and 6B, and S1 Movie**). By analyzing the double-labeled line, we found that the CLoG1-mEGFP accumulation at the apical region of growing cells, at the zone where microtubules overlap at the tip of growing cells. This zone of microtubule overlap has been previously described and shown to be important for polarized growth [43].

**Fig 7:**
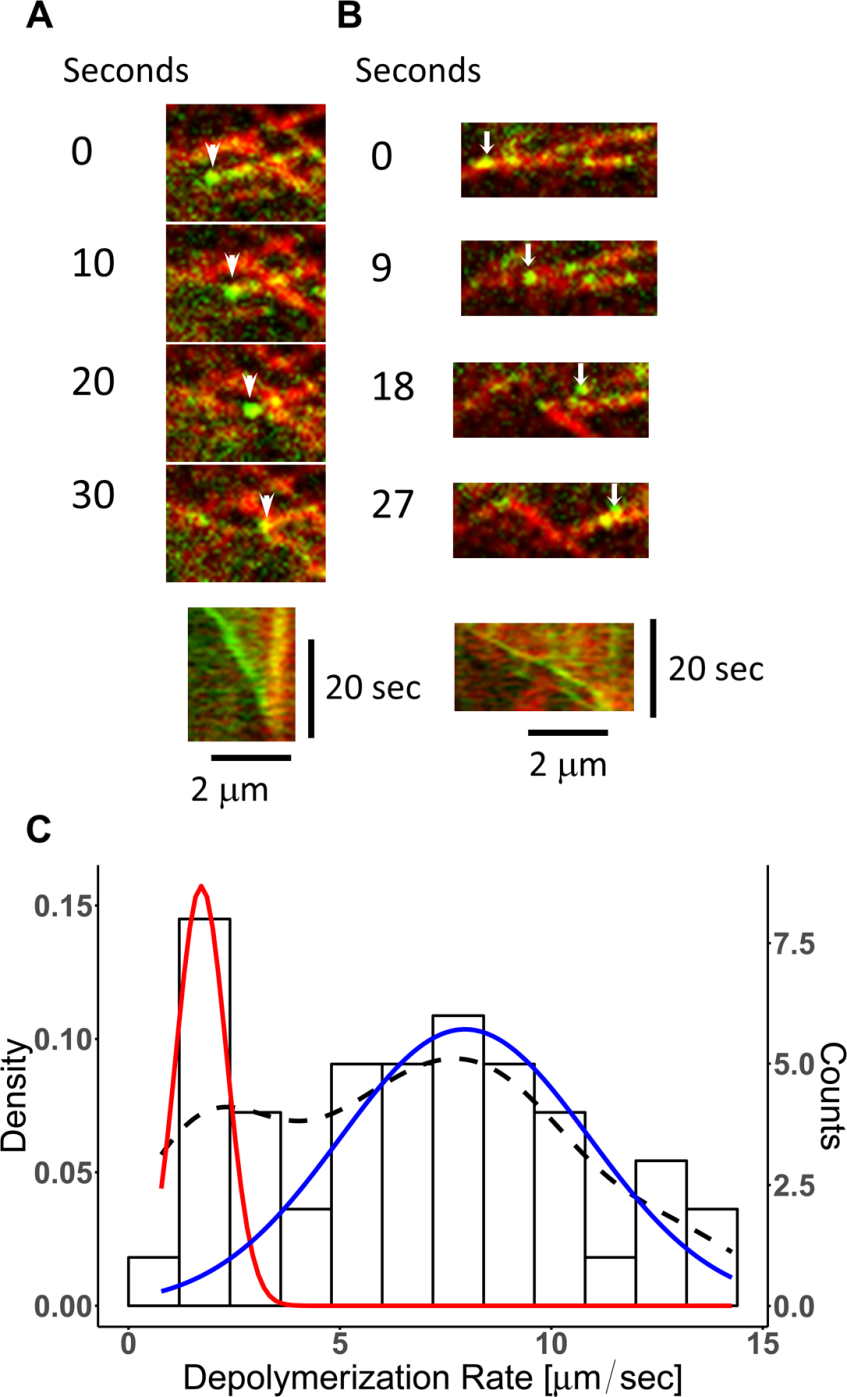
CLoG1 associates with fast and slow microtubule depolymerizing ends. Representative examples of **(A)** slow and **(B)** fast depolymerizing microtubules. CLoG1-mEGFP (green) and mCherry-tubulin (red) were visualized on a subapical cell; arrowheads and small arrows indicate the accumulation of CLoG1-mEGFP at the microtubule’s end. Bottom panel shows the kymographic analysis of the time series. **(C)** Depolymerization rates were estimated by modeling a mix of two Gaussians to the data shown in the histogram. The probability density for both populations is shown with a black dashed line, the individual densities for each population are shown in red and blue lines. The rate values are: slow ends (red) 1.7 ± 0.6 μm/min and fast ends (blue) 7.9 ± 2.9 μm/min (mean ± st. dev.). A total of 46 ends were measured from 3 different cells.

**Fig 8:**
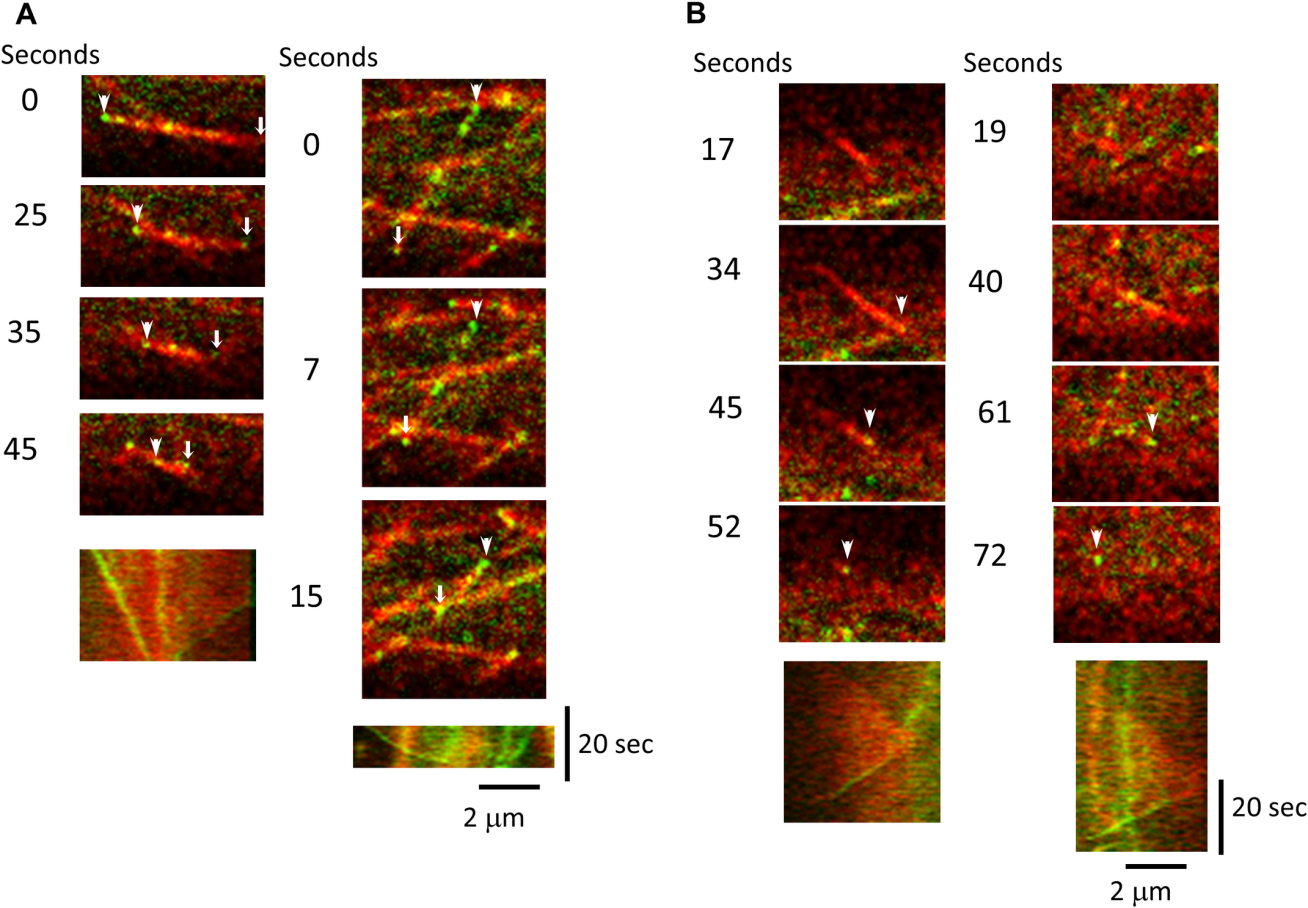
GLoG1 can associate with both ends of a depolymerizing microtubule and with depolymerizing ends after catastrophe. **(A)** Two examples of microtubules undergoing depolymerization from both ends where CLoG1-mEGFP accumulates at both ends. Arrowheads and small arrows indicate slow and fast depolymerization, respectively. Bottom panels show the kymographic analysis of the time series. **(B)** Two examples of microtubules undergoing polymerization followed by catastrophe. Note that CLoG1-mEGFP is not associated with the growing end, but associates with the depolymerization end. CLoG1-mEGFP (green) and mCherry-tubulin (red) were visualized on a subapical cell; arrowheads indicate when GLoG1-mEGFP can be detected after depolymerization starts. Bottom panels show the kymographic analysis of the time series.

Analysis of dividing cells showed an accumulation bias of CLoG1-mEGFP signal toward the spindle poles during anaphase (**Fig 6C, S7 Fig, and S2 Movie**). It is well established that tubulin subunits in mitotic spindles undergo flux with subunit depolymerization at their poles [44, 45]. This accumulation pattern suggested that CLoG1 might be tracking depolymerizing microtubules. To investigate this possibility, we analyzed individual cortical microtubules using high-resolution confocal microscopy of subapical cells, where the cytosolic signal of CLoG1-mEGFP is lower than in apical cells, resulting in an increase of the signal to noise ratio. This analysis confirmed that CLoG1-mEGFP localization on microtubules is punctate, with a bias towards depolymerizing ends (**S3 Movie)**. Kymographs of single depolymerizing microtubules confirmed the accumulation with depolymerizing ends (**Fig 7 and S4–S6 Movies**). The fluorescent protein signal can be detected as a spot on both, slowly (**Fig 7A and 7C and S4 Movie**) and rapidly (**Fig 7B and 7C and S5 Movie**) depolymerizing microtubules. This suggests that CLoG1 can track the plus and minus ends of microtubules during depolymerization. In fact, this double localization is clearly observable in time-lapse movies where both ends of a single microtubule depolymerize simultaneously (**Fig 8A and S6 and S7 Movies**). We never observed accumulation of CLoG1 on growing microtubules, but in some occasions, we were able to observe accumulation on microtubule ends after they stop polymerizing and start depolymerizing (undergoing catastrophe) (**Fig 8B and S8 and S9 Movies**).

### Microtubule dynamics are slower in *CLoG1* knock-down cells

**Fig 9:**
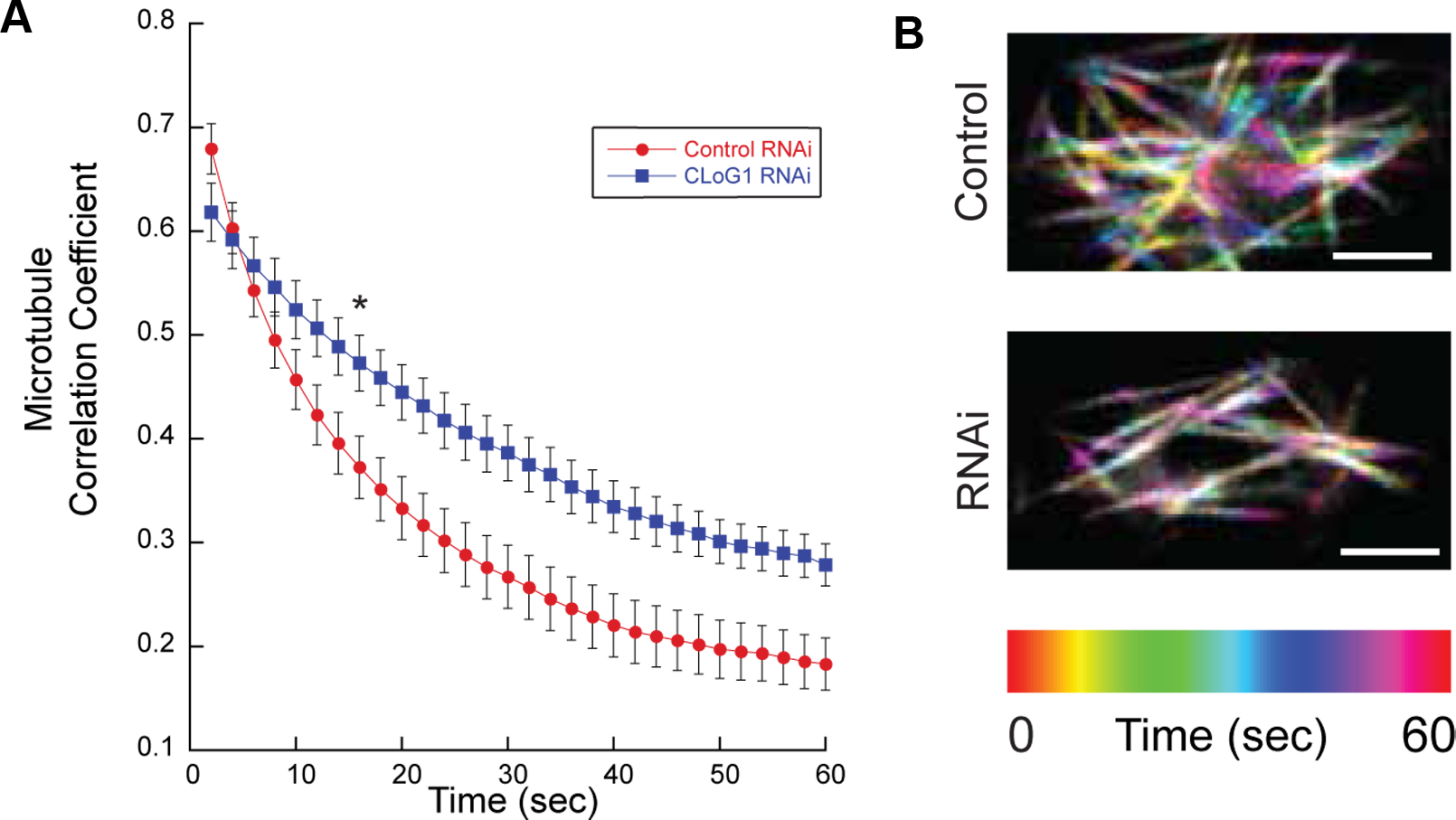
Microtubule dynamics are slower in CLoG1-RNAi cells. **(A)** The microtubule correlation coefficient as a function of time shows that microtubule activity is higher in control RNAi (red) than CLoG1 RNAi (blue) cells, as seen by the more rapid rate of decay. Experiments were done at room temperature. Error bars are standard error of mean. Across two experiments, n = 21 cells for control RNAi and n = 20 for CLoG1 RNAi; two-tail t-tests were done on each time point, and the asterisk at 14 seconds indicates where p<0.05, all time points after maintain significance. **(B)** Temporal-Code Color of representative movies. Scale bar equals 3μm.

CLoG1-mEGFP localizes to microtubules and tracks their depolymerizing ends; therefore, we hypothesized that microtubule dynamics may be altered with reduced levels of CLoG1 protein. To observe microtubule dynamics, we transiently silenced CLoG1 with the CLoG1-UTR-targeting RNAi construct in the background of a line stably expressing mCherry-αTubulin as well as the nuclear silencing marker (NLS-GFP-GUS) [46]. *P*. *patens* protonema have two microtubule populations- cortical and cytoplasmic. We acquired single-plane confocal images near the cortical microtubules of seven-day-old plants to get minimal background images for analysis. To quantify the cortical microtubule dynamics, we measured the correlation coefficient across frames for each movie, as described previously for actin [46, 47] and microtubules [41]. In cells with silenced CLoG1, the correlation coefficient did not decay as rapidly as in control cells, and thus these plants have slower microtubule dynamics (**Fig 9**). The detailed changes in dynamics from individual microtubules were not further analyzed due to difficulty generating a large data set and due to the small cell size of the CLoG1-RNAi cells.

## Discussion

Here we established a strategy to isolate and map loss-of-growth mutations in the moss *P*. *patens*, which enabled identification of a novel and ancient gene important for cell growth. *CLoG1* is conserved in all plants and encodes a protein that localizes to the microtubule cytoskeleton and can track depolymerizing microtubule ends. We accomplished these advances by performing a conditional mutant screen, which is a powerful tool used in fungal and invertebrate systems, to identify genes important for cell growth. We then used segregation analysis and next-generation whole-genome sequencing to map the causal mutation. Taking advantage of efficient homologous recombination in moss, we used genetic complementation to validate the identity of the mutant gene. Finally, we used transient RNAi and functional complementation with mEGFP fusions to demonstrate that the novel protein localizes to microtubules, tracks the depolymerizing ends and may play a role in increasing microtubule dynamics.

Understanding the precise role that *CLoG1* plays in the cell will require further analysis; nevertheless, based on the loss-of-growth phenotype observed and its intracellular localization, we hypothesize that it may play a role in the regulation of microtubule organization during cell division and cell expansion [26, 48, 49]. Our measurements of growing cells shows reduced cell length and increased in cell diameter; these observations are consistent with CLoG1 participation in tip growth and the polarization machinery. Because we did not observe multinucleated cells in the *clog1* cells at the restrictive temperature, we do not expect CLoG1 to play an essential role in cell division, but the number of cells per plant appears to be reduced in the *clog1* plants grown at the restrictive temperature, suggesting that the timing of cell division may be slower in *clog1* cells.

Concerning the effects on microtubule dynamics, the following two possible scenarios could result in the slower dynamics we observed in the CLoG1-RNAi lines: a decrease in the rate of polymerization or depolymerization, or a decrease in the frequency of rescue or catastrophe. Because CLoG1 does not appear to localize to polymerizing ends, we suggest it may increase depolymerization rates. However, it may also play a subtler role by affecting the frequency of catastrophe or rescue events. Detailed analyses of CLoG1 localization with microtubules in the wild type and mutant backgrounds as well as *in vitro* studies should help to distinguish these possibilities.

Consistent with a defect in microtubule dynamics, our localization studies show that CLoG1 accumulates on microtubules. While we expressed the CLoG1-mEGFP protein fusion from a constitutive promoter in the wild type background, it is possible that some of the observed localization is due to over-expression. Thus, additional studies using lines tagged at the endogenous locus with tandem mEGFP tags to boost the fluorescent signal are needed. Nevertheless, it is interesting to note that the observed tracking of depolymerizing plus and minus ends is similar to that of microtubule depolymerizing kinesins, such as the kinesin 13 family [50]; a possible hypothesis is that CLoG1 may associate or regulate kinesin-based end depolymerization. Many microtubule end-binding proteins have been reported previously, including proteins associated with depolymerizing ends [51], but with the exception of kinesins mentioned above, these proteins associate only with one end of the depolymerizing microtubule. Hence, elucidating the mechanism for association of CLoG1 to both depolymerizing ends is likely to reveal a novel and important system for regulating microtubule dynamics during cell division and cell growth during interphase. The identification of CLoG1 as a component of this system will facilitate its analysis by providing a handle for future research.

Our study reveals the great potential of *P*. *patens* in forward genetics that was envisioned by the pioneers of this system [1, 2], and similar to *Arabidopsis*, it guarantees to provide novel insights into many plant biology problems when combined with more sophisticated genetic screening strategies [52]. We anticipate that the combination of haploid genetics, simple development, and reduced genetic complexity will continue to strengthen the role of *P*. *patens* as a model land plant to study many aspects of plant growth and development [53, 54].

## Materials and methods

### Moss culture and protoplasting

Except during crossing, all plants used in this study were proliferated on solid PpNH_4_ plates [55] at the designated temperature (15°C, 20°C, 25°C, or 32°C) under a cycle of 16 h light (90 μmol m^-2^ s^-1^) and 8 h dark. Plant tissue was ground with a homogenizer (Power Gen 125, Fisher Scientific) and transferred onto solid PpNH_4_ plates overlaid with cellophane. One week-old moss was harvested and incubated with a cell wall digestive solution (0.5% (w/v) driselase in 8% (w/v) mannitol) for 1 h in order to remove the cell wall. The protoplasts were sieved through 70 μm mesh to remove debris and then centrifuged, after which the pellet of protoplasts was re-suspended in 10 mL 8% (w/v) mannitol and washed twice more.

### UV mutagenesis and mutant selection

The genetic screen performed here (**S1 Fig**) was based on one used for identifying conditional morphological mutations in *Neurospora crassa* [12] and conditions for UV-induced mutagenesis were adapted from a protocol for isolation of gravitropic moss mutants in *Ceratodon purpureus* [30]. Wild type Gransden *P*. *patens* protoplasts were suspended in 1 to 2 mL liquid PpNH_4_ containing 8% (w/v) mannitol and 10mM CaCl_2_ and their concentration was calculated by counting the number of cells in the suspension using a hemocytometer. The protoplasts were distributed onto 90 mm petri dishes containing solid protoplast regeneration medium bottom [PRMB] overlaid with cellophane. Approximately 500,000 protoplasts were distributed onto each plate, which were then irradiated using UV light (Fisher Scientific FB-UVXL-1000 UV Crosslinker). After regeneration, this resulted in approximately 500 plants per plate. The plates were then incubated at 25°C for four days followed by 32°C for a week. After this, mutant plants were re-suspended in 12 mL sterile liquid PpNH_4_ medium and selected by sieving through a 200 μm nylon mesh. Selected mutants were re-plated at 32°C for another week. Plants with mutant phenotypes were identified by eye, picked with tweezers to a fresh PpNH_4_ plate, cultured at 25°C on a PpNH_4_ plate until the plant reached ~5 mm in diameter, and tested for temperature-sensitivity by grinding and expanding each line on two PpNH_4_ plates: one at 25°C and one at 32°C.

### Growth assay

The growth assay in this study is a modified version of the method described previously [31]. Protoplasts of the control and TS mutants were suspended in 1 to 2 mL 8% (w/v) mannitol and the cells from this suspension were counted using a hemocytometer. Each mutant’s protoplasts were re-suspended in 2 mL melted PRMT medium [55] and kept at 47°C in concentrations of 25,000 and 50,000 cells/mL. This medium was distributed onto 90 mm PRMB plates overlaid with cellophane, on which the protoplasts were regenerated at 25°C for 4 d. After this, the cellophane from each plate was cut into three equal pieces, each of which was transferred to a different PpNH_4_ plate (on Day 0); the three pieces were incubated at 20°C, 25°C, and 32°C.

On day three, microscope slides were prepared by adding 30 μL of 10 μg/mL calcofluor (Fluorescent Brightener 28, Sigma) diluted in distilled water onto a glass slide and inverting a coverslip-sized square of the sample–the cellophane with PRMT agar on top–onto the calcofluor. The cellophane was removed from the agar, another 20 μL calcofluor was added, and a coverslip was placed on top and sealed with a 1:1:1 mixture of melted vaseline:lanoline:paraffin. Imaging was performed with a 10X objective using a Zeiss Axiovert 200M microscope fitted with a CoolSNAP fx CDD camera. Zeiss Axiovision software was used to create an overlapping grid pattern of 200 to 300 pictures and an ImageJ macro [31] was used to measure parameters of the plants in these pictures, such as plant area and solidity. This procedure was repeated three times to reach the sample size indicated in the figure legend (**Fig 1**).

Plant area was normalized to the average area of the wild type plants at either 25°C or 20°C. For statistical comparisons, the variance between experiments and groups has been previously shown to be similar [37]; the area was further normalized by obtaining the natural logarithm, because of the log normal distribution of plant areas [37]. To determine if there was a significant difference in plant area and solidity between the mutants and controls when grown at 20°C, 25°C, and 32°C, OriginPro 8.1 was used to conduct one-way ANOVA-Tukey tests to reject equivalence of means. From these tests, adjusted p-values for comparing *ln*(area) and solidity for 20°C vs. 25°C, 20°C vs. 32°C, and 25°C vs. 32°C were obtained for the mutant line and wild type at day three following transfer to growth medium. If the adjusted p-values were smaller than 0.05, it was assumed that the difference in *ln*(area) or solidity was statistically significant.

To evaluate stable complementation analysis, the growth assay described above was slightly modified. Images were acquired using the MosaicX module from AxioVision that allows for tile-based acquisition and stitching. Composite images were constructed by 10x10 individual images. A single composite image was generated for each condition and analyzed as indicated above. Plants from three experiments were used for the analysis. Statistical comparisons (one way ANOVA-Tukey) and plotting were performed using RStudio.

### Expression of GFP-NLS in the *clog1* background and cell size analysis

The clog1 line and the corresponding wild type lines were transformed with the NLS-GFP-GUS construct previously described [32] and lines expressing similar levels of nuclear GFP signal were selected. For cell size analysis, protoplasts were plated and regenerated in the same conditions as for the growth assay (see above) and analyzed three days after transfer to growth medium. Cell walls were stained with 30 μL of 10 μg/mL calcofluor and the cells were visualized with a Zeiss Observer, 10x lens, DAPI and FTIC fluoresce filters, and equipped with an Apotome for three-dimensional sectioning. Z-stacks were projected by maximal intensity in blue and green channels and pseudo-colored green (wall) and red (nuclei). Length and thickness were determined using the ImageJ measuring tool. In total 40 cells were measured from 3 independent experiments. Statistical comparisons (one way ANOVA-Tukey) and plotting were performed using RStudio.

### Moss crossing

The method used for crossing moss was adapted from standard protocols for identification of hybrid *Physcomitrella patens* sporophytes [33]. TS mutants and fluorescently-labeled *P*. *patens* Villersexel (Vx::mCherry) were proliferated and harvested for crossing at one week old. A special solid medium, BCD medium with low nitrogen [33], was used to help sporophyte development. Deep petri dishes were prepared using 90 mL of this melted medium.

Plant tissue of all the mutants and the polymorphic Villersexel strain was ground with a homogenizer (Power Gen 125, Fisher Scientific), and the ground tissue of each TS mutant was mixed with the same amount of ground tissue of Vx::mCherry. The mixed moss tissue was grown at 25°C for 3 weeks, after which the plates were cultivated at 15°C. After 2 weeks, sterile distilled water was added to each plate to just submerge the tissue and the water was removed after one day. The same procedure was repeated after 3 weeks of culture at 15°C, and sporophytes were picked when capsules turned brown. To identify crossed sporophytes, tissue was observed using a fluorescence stereo microscope (Zeiss) with green light excitation and red light emission filters. The sporophytes of plants with fluorescent capsules on non-fluorescent gametophytes were chosen.

One to three sporophytes were harvested in a sterile 1.5 mL microcentrifuge tube and then sterilized following published protocols [56]. To germinate the spores, the capsules were gently crushed with the pipette tip and mixed to produce a spore suspension, and approximately 400 μL of this suspension was distributed evenly onto germination solid medium in 90 mm petri dishes. The germination medium recipe is available from PHYSCObase’s spore germination protocol (moss.nibb.ac.jp). When plants were large enough, they were picked onto PpNH_4_ agar plates. To screen for F1 segregants that retained the TS phenotype, each segregant and a control plant were proliferated on two PpNH_4_ agar plates each and incubated at 25°C and 32°C for one week. Imaging was performed on a stereomicroscope under white light at a magnification of 64X and because this selection was qualitative, only segregants that could be clearly identified as temperature-sensitive were pooled.

### Genomic analysis

#### Monte Carlo simulation

To estimate how the size of the mapping interval (the possible region containing the causal mutation) changes with respect to the size of mapping population (number of F1 segregants), a Monte Carlo simulation was conducted using MATLAB (code available upon request). The size of the mapping interval was defined as the size of chromosomal segment(s) where no crossover occurred. In order to simulate the crossover process, a few assumptions were made about the crossover process: 1) there is one causal mutation (SNP) located randomly on one nucleotide of any of the 27 chromosomes (based on known characteristics of UV-induced mutations and TS mutations and also the unclear locations of telomeres and centromeres) [13, 36, 57, 58]; 2) there is either zero, one, or two crossovers per chromosome (based on a study of the crossover landscape of outcrossed F2 *Arabidopsis* which found this to be the case approximately 70% of the time) [34]; 3) the average number of crossovers of a certain chromosome is positively correlated with the chromosome’s length (based on the crossover landscape of F2 *Arabidopsis*) [34]; 4) if there is only one crossover on a chromosome, the crossover point is randomly chosen from all nucleotides of that chromosome (i.e., no influence from centromere or telomeres); and 5) if there are two crossovers on a chromosome, the crossover point of the first crossover is random, and the distance between the second crossover and the first is generated according to a gamma distribution whose shape and scale parameters are correlated with the chromosome’s length [34].

The chromosome lengths of *P*. *patens* (**S2 Table**) were obtained from the V3 genome assembly (*Physcomitrella patens v3*.*0 early release*), and the chances of zero, one, and two crossovers per chromosome and the shape and scale parameters of gamma distributions were calculated accordingly (**S3 and S4 Tables**). The Monte Carlo simulation was formulated to generate random crossovers for a defined number of F1 segregants of *P*. *patens* based on the aforementioned five assumptions and to determine the distribution of the sizes of mapping intervals by repeating the random crossovers for a defined number of F1 segregants 100 times. The simulation’s algorithm followed these steps: 1) randomly select the causal mutation for a population of a defined number of mutants; 2) for each chromosome of each F1 segregant, determine the number of crossovers according to its chances of having zero, one, or two; 3) determine crossover location(s) for chromosomes with one or two crossover(s) randomly or according to pre-calculated gamma distribution; 4) determine how chromosomes are segregated into F1 segregants, assuming random segregation and selection for causal mutation; 5) update the mapping interval with known crossover locations; 6) repeat steps two to four for the defined number of mutants in a mapping location; 7) count the number of chromosomes and base pairs in the mapping interval; and 8) repeat steps one to seven 100 times and record the results. This simulation was run for a population size of 10, 20, 24, 50, 70, and 90 in order to find the lower limit of for a reasonably-sized mapping population and to find how the size of the mapping interval decreases with increasing size of the mapping population. The results of this simulation are shown in **S2 Fig**.

#### Genome sequencing of pooled segregants

Based on the results of the simulation and of existing studies that used genome sequencing of pooled segregants to map randomly-generated casual mutations [6, 7], the size of the mapping population, sequencing depth, and sequencing type were decided to be 24, 10X coverage, and paired-ended with 90 nucleotide read length, respectively. In order to extract approximately the same amount of genomic DNA from each of the 24 F1 segregants of TS mutant *clog1*, approximately 0.2 g of one-week old moss was weighed for each segregant. The genomic DNA of these samples were then extracted using the Mo Bio PowerPlant Pro DNA Isolation Kit in groups of two. The concentration and purity of each DNA extraction were measured using a NanoDrop 2000c UV-Vis spectrophotometer (Thermo Scientific), and equal amounts of DNA from all extractions were pooled together. DNA was sequenced using next-generation sequencing (Illumina Hiseq^TM^200, conducted by Beijing Genomics Institute, http://www.genomics.cn/en/index). DNA sequencing yielded 5,536,364,400 bp, using paired-end reads (500bp insert size, 90bp read length) where 96.2% of the reads had quality score higher than 20. The quality score (sQ) is calculated using Eq 1 below where E stands for sequencing error rate. The high quality reads were aligned to the V3.0 reference genome using Burrows-Wheeler Aligner (BWA) version 0.7.8—the mem algorithm with default settings. The alignment was of good quality with 48,573,929 out of 61,515,160 (79%) high-quality reads aligning to the genome.

$$sQ=-10{log}_{10}E$$ (1)

#### Causal mutation mapping

Dense SNP markers across the whole-genome are required for analyzing the crossover landscape of pooled segregants. Therefore, a set of SNP markers was obtained by comparing the genomic sequence of *P*. *patens* Gransden and *P*. *patens* Villersexel. Since the Villersexel genome assembly was unavailable, the V3 genome assembly of Gransden (*Physcomitrella patens v3*.*0 early release*) was used as the reference sequence in the alignment of Villersexel sequencing reads (Joint Genome Institute, 2013). The alignment was performed using BWA [59] and variant calling was conducted using SAMTOOLS [60] to generate SNP markers. The alignment was processed with default options. When generating a pileup file for variant calling using SAMTOOLS, the C parameter was set to 50 for downgrading mapping quality of reads, and the thresholds of map quality and base quality were set to 30 while no indel was called. At the end, the SNPs were again filtered by vcfutils.pl varFilter (a subprogram of SAMTOOLS) to only keep those with root mean square (RMS) mapping quality higher than 30, read depth more than five but fewer than 200, and at least three reads supporting the alternate base.

The sequencing reads of *clog1* segregants were then analyzed with the same pipelines but slightly adjusted command options to detect occurrence of recombination at the SNP marker positions. The reads were aligned to the *P*. *patens* V3 genome assembly. To increase the sensitivity of SNP detection, the–E option was applied instead of setting the C parameter to 50 in the generation of a pileup file while other options were kept the same as analyzing the Villersexel genome. After the SNPs at the marker positions were called, they were again filtered by vcfutils.pl varFilter to only keep those with root mean square (RMS) mapping quality higher than 30, read depth more than five but fewer than 80, and at least one read supporting the alternate base.

For the mapping of the causal mutation, the reference allele (Gransden) frequency at the marker positions, marker density, and average read depth were calculated using sliding windows of 40 Kbp for each of the 27 chromosomes. The reference allele frequency is equivalent to the number of reads consistent with the Gransden allele divided by the total read depth at a specific marker position. The marker density is equivalent to the total number of SNPs within the 40 Kbp window. The average read depth is equivalent to the total read depth of SNPs detected in the pileup file of the pooled segregants divided by the number of SNPs within the 40 Kbp window. The rest of the genome’s scaffolds were not considered in this analysis due to their small sizes. The reference allele frequency, marker density, and average read depth were plotted against chromosomal positions for all 27 chromosomes using a simple moving average model with a window size of seven. All calculations and visualizations were conducted using MATLAB (code available upon request).

A 1 Mbp segment centered at the peak of reference allele frequency was selected as the region where the causal mutation was likely to be located. The candidate causal mutations were selected by filtering for non-synonymous and non-marker SNPs in the genes located in the 1 Mbp segment. Since genome annotation files were only available for the V1.2 genome assembly [61], the sequencing reads of pooled segregants were also aligned to the V1.2 assembly. The pipelines and options used for alignments to V3 and V1.2 genome assemblies and variant calling were the same except that the variant calling for alignment to V1.2 assembly was not limited to marker positions. The detected SNPs were then annotated with SnpEff [62] and the *P*. *patens* V1.6 genome annotation [63]. The Gbrowser on Phytozome.org provided the cross reference of V3 and V1.6 genome annotations, and the V1.6 annotation of all genes located in the 1 Mbp segment was found.

### Verification of *CLoG1* identity via genetic rescue

We confirmed the presence of the *clog1* mutation, mapped and identified as indicated above, by amplifying and sequencing the region of the locus predicted. Primers CLoG1-mut(F) and CLoG1-mut(R) (**S5 Table**) were used to amplify the DNA region containing the mutation from wild type (Gransden) and *clog1* plants; primers CLoG1-inF and CLoG1-inR were used for sequencing the PCR product. To confirm the causal nature of this mutation via genetic rescue, plant DNA was isolated from wild type *P*. *patens* (Gransden) using the PowerPlant Pro DNA Isolation Kit (Mo Bio). DNA was amplified using two rounds of PCR reactions with the primers CLoG1-mut(F) and CLoG1-mut(R), and the PCR product purified (NucleoSpin Extract II kit Machery-Nagel); the total PCR product yield was ~30 μg. This wild type-derived PCR product, together with pTH-Ubi-3XmEGFP (used for transient selection of hygromycin-resistant plants), were transformed into *clog1* mutants following standard transformation procedures [55]. Nineteen plants resulting from the transformation were expanded and DNA was extracted as above. PCR was performed for each sample extracted from these plants using the external primers CLoG1-outF and CLoG1-outR. These primers were selected external to the previous set to amplify the putative CLoG1 locus and to avoid amplification of any unintentional insertion site of the previous targeting PCR product. PCR products amplified with primers CLoG1-outF and CLoG1-outR were gel-purified and sequenced using primers CLoG1-inF and CLoG1-inR.

To confirm that that the background of the mutant line (*clog1*) was present in the rescued plant, and that we were not analyzing accidentally a wild type plant, we identified and amplified a mutation found elsewhere in chromosome 24 of *clog1* but not in the wild type plants. This mutation was identified using MATLAB code designed for comparing differences in nucleotides between the sequenced pool of DNA and the wild type genomes (code available upon request). We identified one mutation present in the *clog1* background but absent in the wild type genomes (Gransden and Villersexel). To avoid any interference from the rescue DNA, the mutation was located at position 10,809,758 of chromosome 24, which is several Kb from the putative casual mutation. Primers mutCLoG1-2F and mutCLoG1-2R were designed to bind between positions 10,809,479 and 10,810,028 generating a 550 bp PCR product. Following amplification and purification, the PCR product was sequenced using the same primers used for amplification. The phenotype of rescued and mutant plants was compared using the growth assay indicated above. The procedure was repeated three times to reach the sample size indicated in the figure legend (**Fig 4**).

### Amino acid sequences alignment and phylogenetic tree construction

Protein sequences for CLoG1 protein homologues were identified by BLAST (default settings) in the Phytozome web portal (phytozome.jgi.doe.gov) or the ENTREZ web portal (blast.ncbi.nlm.nih.gov). In most cases only a single gene locus was identified. This is consistent with results from the Panther Classification System for gene families (www.pantherdb.org). All sequences analyzed and the corresponding accession numbers are listed in **S6 Table**. Multiple alignment and gene construction were done using the Geneious R7 software. Multiple alignment was done with the ClustalW algorithm using default settings. A maximum likelihood tree was obtained with the PHYML plugin [64], using the Le Gascuel substitution model and bootstrap for branch support (100 bootstraps). A consensus tree was generated where only branches having more than 50% support are shown. Hydrophobicity and secondary structure plots were also determined using the Geneious default settings. To identify highly conserved regions in CLoG1, an alignment of two amoeboid protists, two algae, and two land plant proteins sequences was performed and an identity graph displaying a 30 residue window was used to visually identify conserved regions.

### Transient silencing of *CLoG1* and complementation of *CLoG1* 5´UTRi

To observe a silencing phenotype of CLoG1, a silencing construct was designed to target a 500 bp region of the 5´ untranslated region (5´UTR) of CLoG1. This region was PCR amplified from wild type (Gransden) cDNA using primers CLoG1UTRi500bpF and CLoG1UTRiR (**S5 Table**) and cloned into pENTR/D-TOPO. After sequencing, we used an LR clonase reaction (Invitrogen) to transfer the amplified region into the silencing vector pUGGi [65]. The resulting construct was named CLoG1-UTRi. Thirty μg of CLoG1-UTRi silencing construct was transformed into a line stably expressing GFP-GUS with a nuclear localization sequence (NLS-4) [65], as previously described [37, 38, 47, 58, 66]. Briefly, antibiotic resistant plants are visually selected for the loss of nuclear GFP signal, which indicates they are actively undergoing gene silencing of the target gene (in this case CLoG1). The plants are photographed using the chlorophyll out fluorescence and their area and solidity (convex hull area/area) are calculated for statistical comparison. Phenotypes were observed and measured on 7-day-old plants. To rescue the silencing of CLoG1, the wild type CLoG1 coding sequence was PCR-amplified from cDNA using primers CLoG1-full-cds-F and CLoG1-full-cds-R. The PCR product was then cloned into pENTR/D-TOPO, and the coding sequence was inserted into an expression vector, pTHUBI-gate [37], via an LR clonase reaction (Invitrogen). The resulting construct was named pTHUbi-CLoG1cds. The mEGFP:CLoG1 fusion constructs were created using Invitrogen Multisite Gateway Pro 2.0 kit. For the C-terminal fusions, CLoG1 cDNA was PCR amplified using primers attB1CLoG1F and attB5rCLoGR, to produce attB1 and attB5r-flanked CLoG1 cDNA. The flanked PCR fragment was cloned into pDONR P1-P5r vector via a BP clonase reaction (Invitrogen). This entry clone was sub-cloned, along with an entry clone containing mEGFP flanked with attB5 and attB2, into the pTHUBI-gate destination vector using an LR clonase reaction (Invitrogen). For the N-terminal fusions, the expression clone was constructed using the same method above; however, the CLoG1 cDNA PCR fragment was flanked with attB5 and attB2 while the mEGFP was flanked with attB1 and attB5r. The primers used for the CLoG1 cDNA PCR were attB5CLoG1F. Thirty μg of CLoG1-UTRi and 2.5–15 μg of pTHUbi-CLoG1cds or the pTHUbi-mEGFP:CLoG1 fusions were co-transformed into NLS-4 protoplasts, and phenotypes were observed and measured on 7-day-old plants using the growth assay described above, but using liquid plating medium instead of solid plating medium. This procedure was repeated at least three times to reach the sample size indicated in the figure legend (**Fig 5**).

### Stable expression of fluorescent protein fusions of CLoG1 and confocal microscopy

The pTHUBI constructs containing C and N terminal mEGFP fusions of CLoG1 were linearized using the SwaI enzyme and transformed into wild type moss using PEG-mediated transformation, and stable lines were generated following standard protocols [55]. Plants expressing mEGFP signal were screened by laser scanning confocal microscopy (Leica SP5). Cell lines expressing the lowest detectable level of expression and showing normal morphology were selected for high resolution microscopy. To observe growing cells, the plants were cultured on a thin layer of agar prepared on a coverglass of a Mattek dish [67]. Under these conditions caulonemal cells grow for several days and can be observed with high numerical aperture (NA) optics. To image the cells, we used the 63X 1.4 NA lens of the SP5 system (Leica) upgraded with a hybrid detector. Images were acquired at 0.68–1 sec. intervals. Images were background subtracted with a radius of 30 and contrast enhanced by histogram stretching (normalization) allowing a 0.4% of saturated pixels using ImageJ (Fiji distribution).

A single moss line expressing a CLoG1 with a C-terminal fusion of mEGFP was transformed with the plasmid pTZUbi-mCherry-tubulin that expresses mCherry-labeled alpha-tubulin and selected with Zeocin [40]. Lines expressing the tubulin reporter were selected and analyzed by confocal microscopy. For high-resolution time series acquisition, the confocal pinhole was closed to 0.4–0.5 airy units. Scanning rate was set to 200 Hz, the acquisition format set to 512x246 pixels, with a zoom of 6 and a pixel size of 80.2 nm. To maximize signal acquisition and reduce background a hybrid detector was used to acquire the CLoG1-mEGFP signal. The green channel (CLoG1-mEGFP) was background subtracted (radius of 20) and normalized (0.4% saturation), the red channel (microtubules) was background subtracted (radius 50), filtered with unsharp mask (radius 10, mask weigh 0.5) and Gaussian blur (sigma 1), and normalized (0.4% saturation). Equivalent settings were used to image spindle and phragmoplast formation. For kymographic analysis of microtubule ends, we use the “Multi Kymograph” function of the ImageJ (Fiji distribution). An in-house macro (available upon request) was used to track the microtubule ends from kymographs and determine their depolymerization rates based on the angles formed. To estimate the mean velocities for fast and slow depolymerizing ends we used a two Gaussians mixture model from the mixtools package (normalmixEM procedure) from R (RStudio), which is based on the iterative expectation maximization (EM) algorithm.

### Measuring bulk microtubules dynamics

CLoG1 UTR RNAi plasmid was transformed into a NLS4/mCh-Tubulin line as described above. Both test and control transformants were selected for with hygromycin selection. Seven-day-old plants were visually screened for active silencing (lack of nuclear GFP) via fluorescent stereomicroscope (Nikon SMZ1500), and regions containing plants were marked for confocal imaging. Silenced plants were transferred to an agar pad mounted slide for confocal imaging. Cortical mCh-Tubulin was excited with a 561nm laser, and emission was collected with a 570+nm bandpass. ImageJ was used for post-acquisition processing. The correlation coefficient analysis was performed in MatLab as previously described [47]. Traces for individual cells were compared, and outliers were removed from the final average. An outlier was defined as a cell with a trace that was outside 1.5 times the interquartile range more than 50% of the trace.

## Supporting information

S1 Fig Diagram outlining a genetic screen to identify temperature-sensitive mutants in *Physcomitrella patens*. (PDF)

S2 Fig Relationship between mapping interval size and mapping population size. (PDF)

S3 Fig Maximum likelihood phylogenetic tree based on the amino acid sequence alignment of proteins homologous to CLoG1 from amoeboid protists, green algae, and land plants. The tree is the consensus from 100 bootstrap and only branches with more than 50% support are shown. *P*. *pallidum* was selected as the root. Accession numbers for the sequences are indicated in S6 Table. Scale bar indicates substitutions per site.
(PDF)

S4 Fig Amino acid sequence of CLoG1 with hydrophobicity plot and secondary structure prediction. A sliding window of 5 was used for the hydrophobicity plot (below the sequence, red indicates high hydrophobicity). Secondary structures are blue cylinders for alpha-helices, green arrows for beta-strands, blue arrows for turns, and grey for coils. Secondary structure predicted with the Geneious software is only 65% accurate. Red boxes indicate the most conserved regions (see S5 Fig).
(PDF)

S5 Fig Amino acid sequence alignment of the most conserved regions of CLoG1 homologues. Two amoeboid protists, two green algae, and two land plants were selected for comparison. Regions with identities above ~20% were selected. The largest highly conserved regions are present both at the N and C termini. Note the presence of abundant leucine residues.
(PDF)

S6 Fig Stable complementation of *clog1* mutant by CLoG1-mEGFP. Three lines were tested all showing significant higher levels of growth and cell polarization at the restrictive temperature (32°C) when compared with the *clog1* mutant.
(PDF)

S7 Fig Two additional representative examples of CLoG1-mEGFP bias to the spindle poles during anaphase. Note that CLoG1-mEGFP (green) is present in the whole spindle but accumulates toward the spindle poles in relation to mCherry-tubulin (red). Compare with Fig 6 in the main text.
(PDF)

S1 Table Number of CLoG1 genes present in different fully-sequenced plant and algal genomes. (PDF)

S2 Table Approximate chromosome lengths of *Physcomitrella patens* used in the Monte Carlo simulation. (PDF)

S3 Table Approximate chances of 0, 1, and 2 crossovers(s) on 27 chromosomes of *P*. *patens*. (PDF)

S4 Table Scale and shape parameters of the gamma distribution which determines the distance between the first and second crossovers in the Monte Carlo simulation. (PDF)

S5 Table Primers used in this study. (PDF)

S6 Table Species, accession numbers, and amino acid sequence lengths of CLoG1 protein homologues used in this study. (PDF)

S1 Movie Apical caulonemal cell expressing a C-terminal mEGFP fusion of CLoG1 and mCherry-Tubulin. Scale bar = 5 microns.
(MOV)

S2 Movie Mitosis and cytokinesis of a caulonemal cell expressing a C-terminal mEGFP fusion of CLoG1 and mCherry-Tubulin. Scale bar = 5 microns.
(MOV)

S3 Movie Cortical microtubule and CLoG1 dynamics in a caulonemal cell expressing C-terminal mEGFP fusion of CLoG1 and mCherry-Tubulin. Scale bar = 5 microns.
(MOV)

S4 Movie Short sequences of a representative depolymerizing microtubule illustrating slow end depolymerization and CLoG1 accumulation in a caulonemal cell expressing C-terminal mEGFP fusion of CLoG1 and mCherry-Tubulin. Scale bar = 5 microns.
(MOV)

S5 Movie Short sequences of a representative depolymerizing microtubule illustrating fast end depolymerization and CLoG1 accumulation in a caulonemal cell expressing C-terminal mEGFP fusion of CLoG1 and mCherry-Tubulin. Scale bar = 5 microns.
(MOV)

S6 Movie Short sequence of a representative depolymerizing microtubule illustrating simultaneous two end depolymerization and CLoG1 accumulation in a caulonemal cell expressing C-terminal mEGFP fusion of CLoG1 and mCherry-Tubulin. Scale bar = 5 microns.
(MOV)

S7 Movie Short sequence of a representative depolymerizing microtubule illustrating simultaneous two end depolymerization and CLoG1 accumulation in a caulonemal cell expressing C-terminal mEGFP fusion of CLoG1 and mCherry-Tubulin. Scale bar = 5 microns.
(MOV)

S8 Movie Short sequence of a representative polymerizing and depolymerizing microtubule (catastrophe) illustrating association of CLoG1 to a depolymerizing end in a caulonemal cell expressing C-terminal mEGFP fusion of CLoG1 and mCherry-Tubulin. (MOV)

S9 Movie Short sequence of a representative polymerizing and depolymerizing microtubule (catastrophe) illustrating association of CLoG1 to a depolymerizing end in a caulonemal cell expressing C-terminal mEGFP fusion of CLoG1 and mCherry-Tubulin. (MOV)
