# Supplementary figures and images for: Conditional genetic screen in Physcomitrella patens reveals a novel microtubule depolymerizing-end-tracking protein
Source: PLoS Genet. 2018 May 10;14(5):e1007221. doi: 10.1371/journal.pgen.1007221 (PMC5944918; doi:10.1371/journal.pgen.1007221)

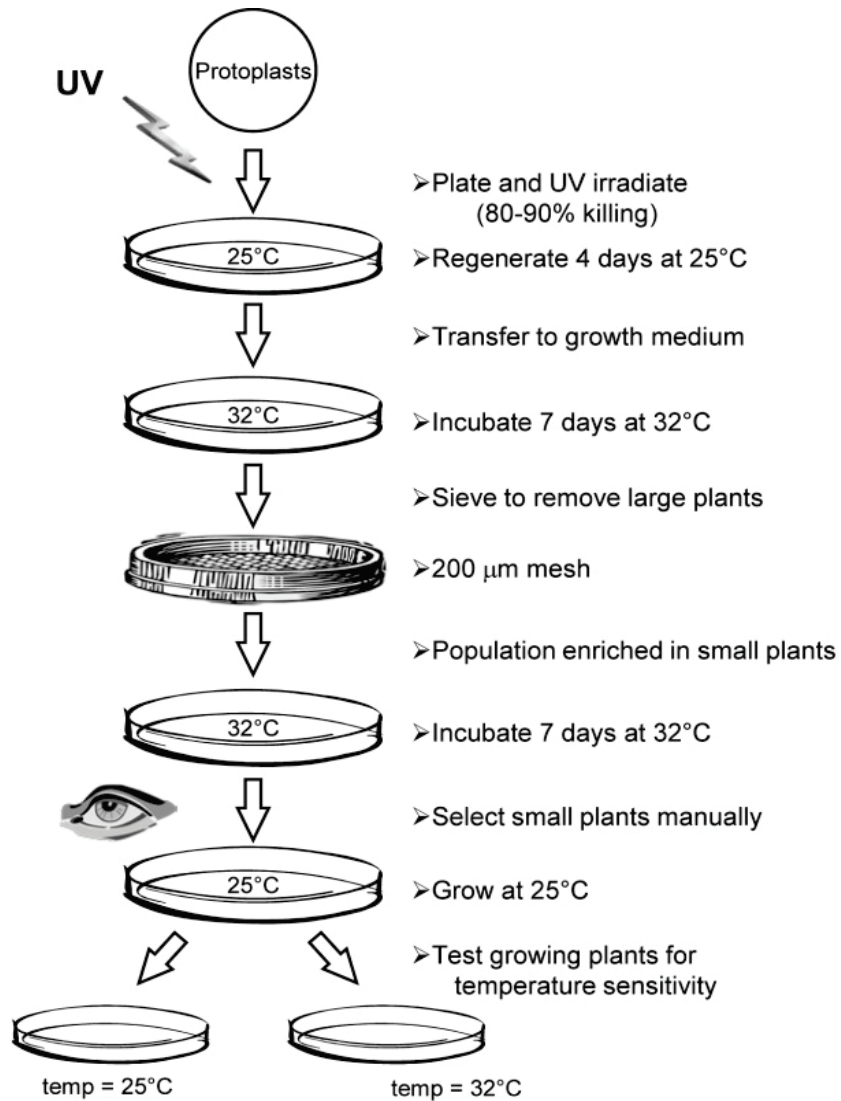

**Temperature sensitive plants will grow well at 25°C but will not grow at 32°C**

Supplement: S1 Fig — (PDF) [file pgen.1007221.s001.pdf]

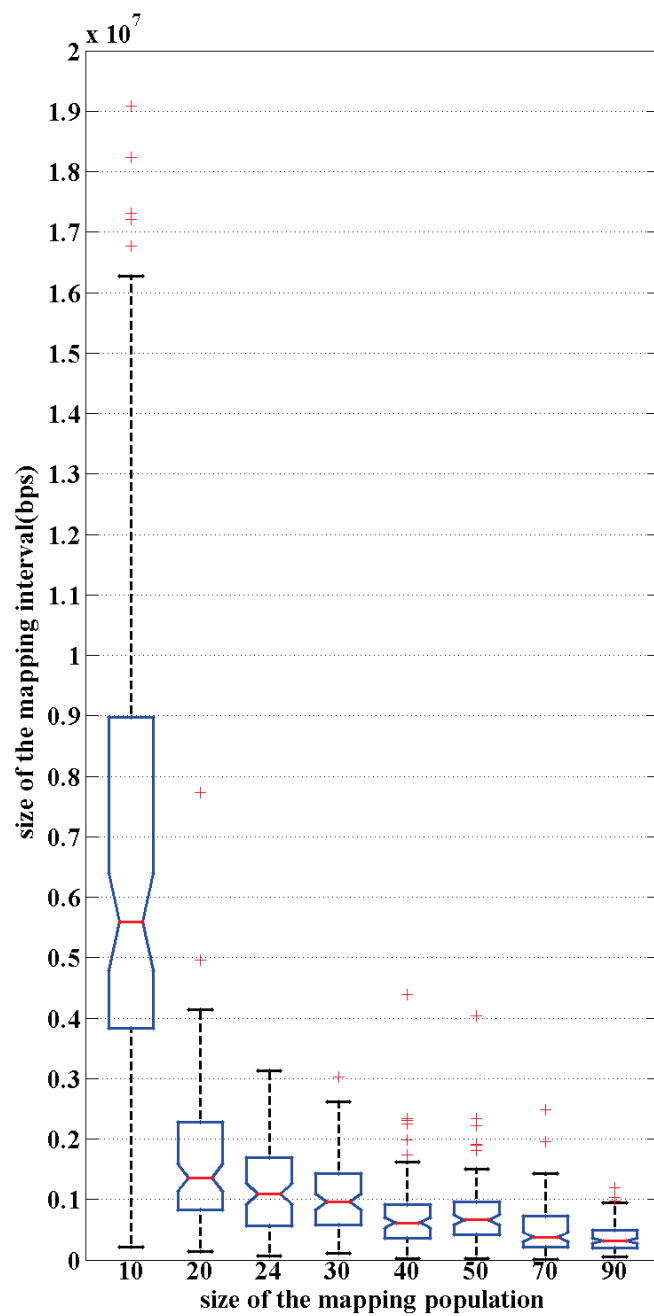

Supplement: S2 Fig — (PDF) [file pgen.1007221.s002.pdf]

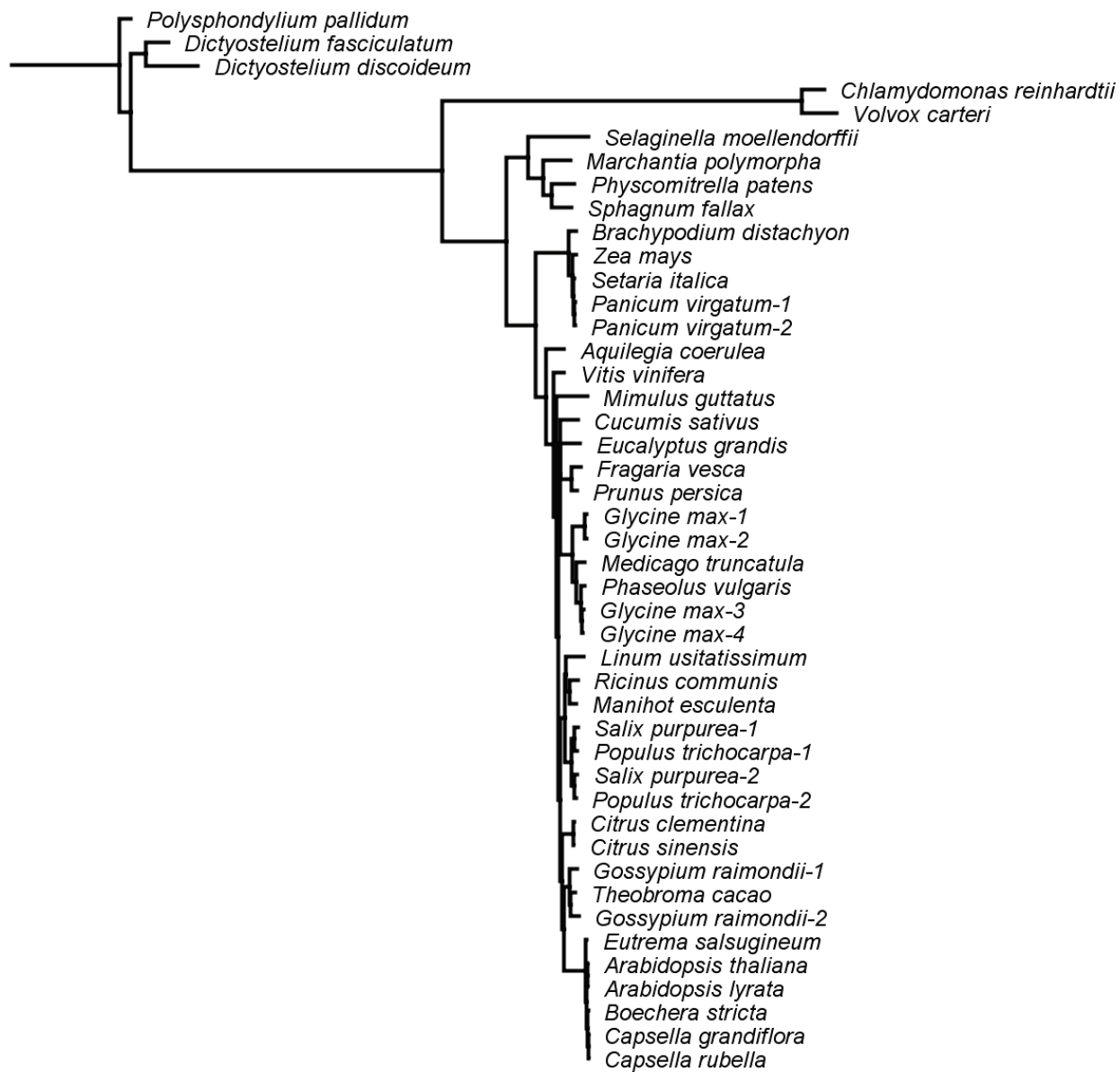

2.0

Supplement: S3 Fig — The tree is the consensus from 100 bootstrap and only branches with more than 50% support are shown. P. pallidum was selected as the root. Accession numbers for the sequences are indicated in S6 Table. Scale bar indicates substitutions per site. (PDF) [file pgen.1007221.s003.pdf]

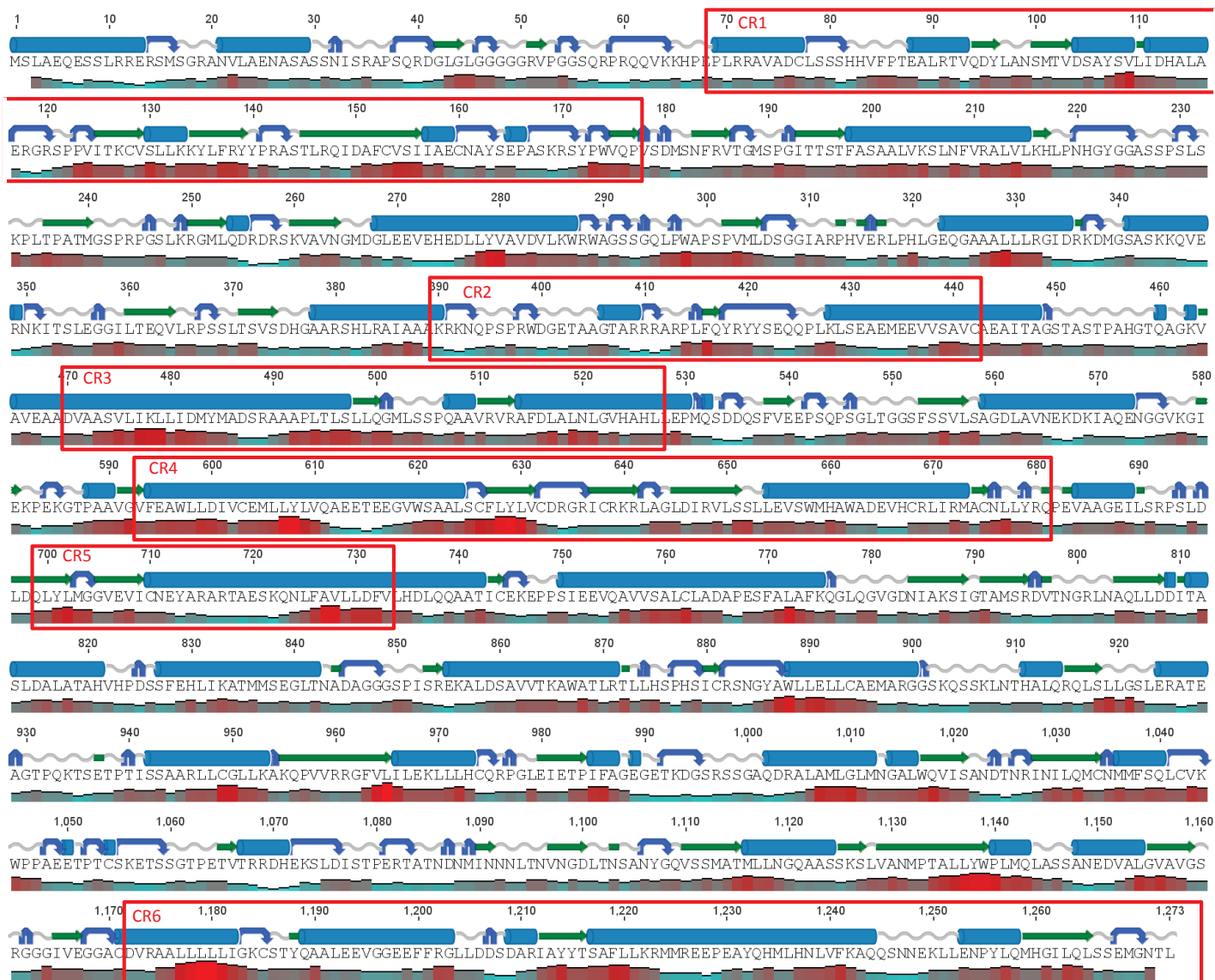

Supplement: S4 Fig — A sliding window of 5 was used for the hydrophobicity plot (below the sequence, red indicates high hydrophobicity). Secondary structures are blue cylinders for alpha-helices, green arrows for beta-strands, blue arrows for turns, and grey for coils. Secondary structure predicted with the Geneious software is only 65% accurate. Red boxes indicate the most conserved regions (see S5 Fig). (PDF) [file pgen.1007221.s004.pdf]

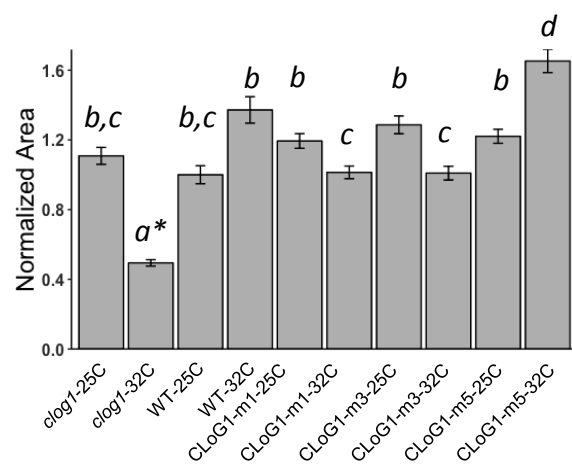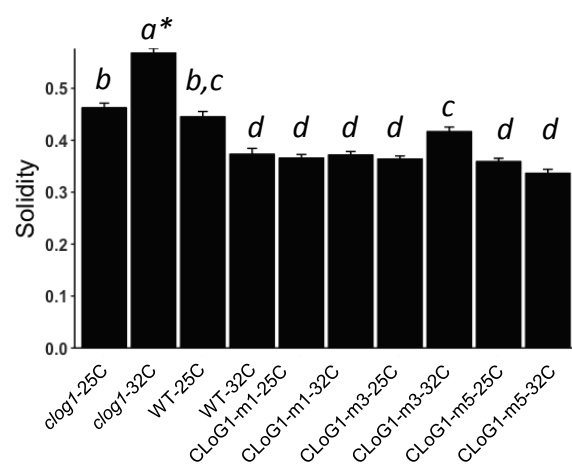

Supplement: S6 Fig — Three lines were tested all showing significant higher levels of growth and cell polarization at the restrictive temperature (32°C) when compared with the clog1 mutant. (PDF) [file pgen.1007221.s006.pdf]

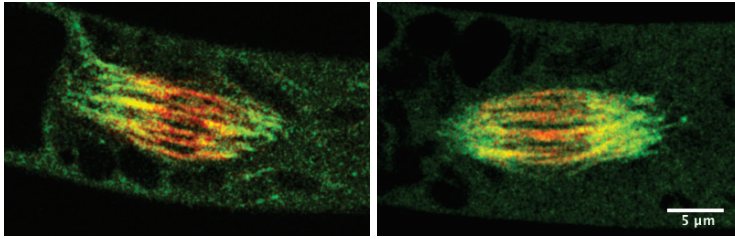

Supplement: S7 Fig — Note that CLoG1-mEGFP (green) is present in the whole spindle but accumulates toward the spindle poles in relation to mCherry-tubulin (red). Compare with Fig 6 in the main text. (PDF) [file pgen.1007221.s007.pdf]
