# Supplementary material for: Conditional genetic screen in Physcomitrella patens reveals a novel microtubule depolymerizing-end-tracking protein
Source: PLoS Genet. 2018 May 10;14(5):e1007221. doi: 10.1371/journal.pgen.1007221 (PMC5944918; doi:10.1371/journal.pgen.1007221)
Supplement: S5 Fig — Two amoeboid protists, two green algae, and two land plants were selected for comparison. Regions with identities above ~20% were selected. The largest highly conserved regions are present both at the N and C termini. Note the presence of abundant leucine residues. (PDF) [file pgen.1007221.s005.pdf]

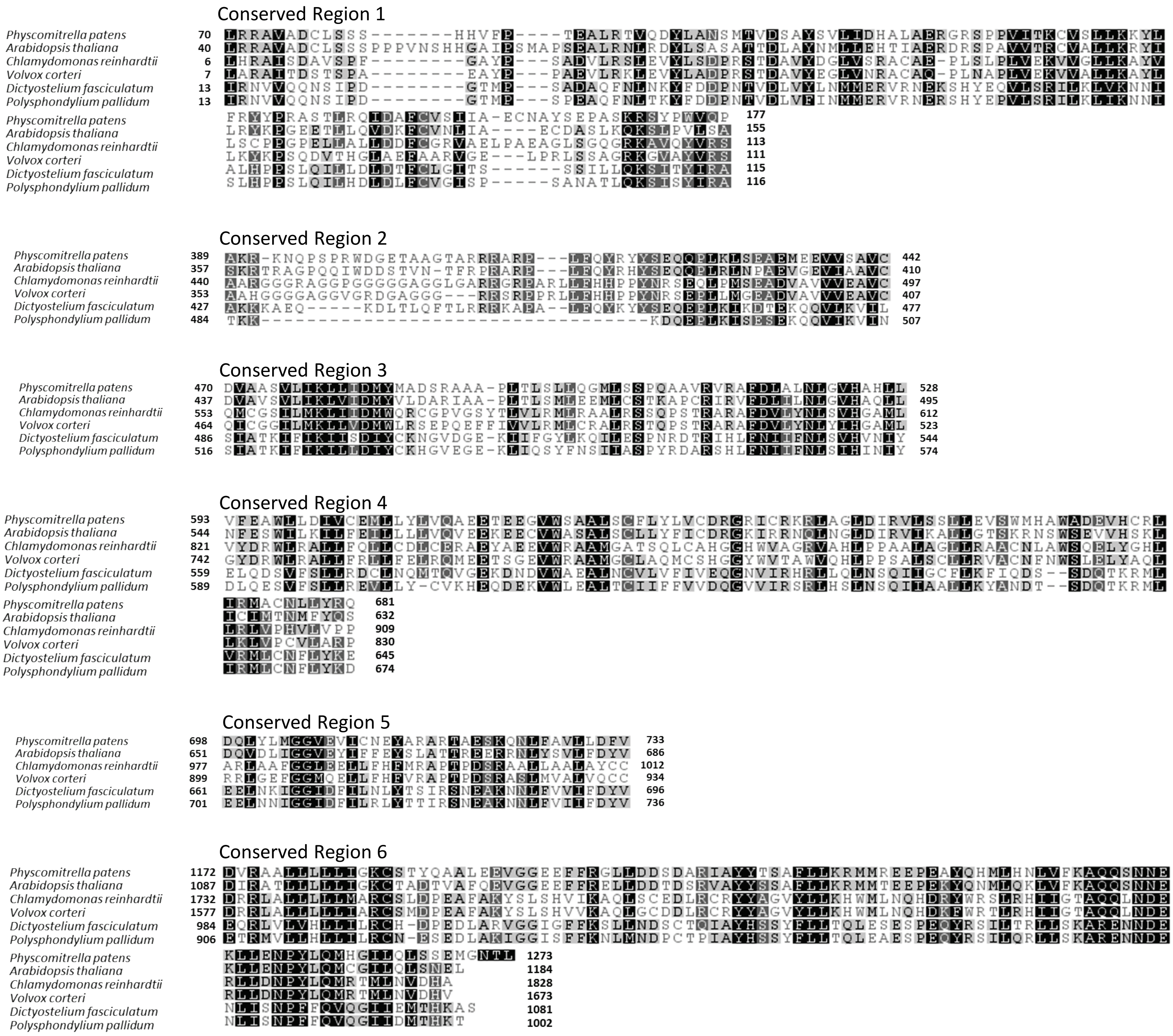
Conserved Region 1
Physcomitrella patens 70 LRRAVADCLSSS-------HHVFP----TEALRTVQDYLANSMTVDSAYSVLIDHALAERGRSPPVITKCVSLLKKYL
Arabidopsis thaliana 40 LRRAVADCLSSSPPPVNSHHGAIPSMAPSEALRNLRDYLSASATTDLAYNMLLEHTIAERDRSPAVVTRCVALLKRYI
Chlamydomonas reinhardtii 6 LHRAISDAVSPF--------GAYP---SADVLRSLEVYLSDPRSTDAVYDGLVSRACAE-PLSLPLVEKVVGLLKAYV
Volvox corteri 7 LARAITDSTSPA--------EAYP---PAEVLRKLEVYLADPRSTDAVYEGLVNRACAQ-PLNAPLVEKVVALLKAYL
Dictyostelium fasciculatum 13 IRNVVQQNSIPD--------GTMP---SADAQFNLNKYFDDPNTVDLVYLNMMERVRNEKSHYEQVLSRILKLVKNNI
Polysphondylium pallidum 13 IRNVVQQNSIPD--------GTMP---SPEAQFNLTKYFDDPNTVDLVFINMMERVRNERSHYEPVLSRILKLIKNNI
Physcomitrella patens FRYYPRASTLRQIDAFCVSIIA-ECNAYSEPASKRSYPWVQP 177
Arabidopsis thaliana LRYKPGEETLLQVDKFCVNLIA----ECDASLKQKSLPVLSA 155
Chlamydomonas reinhardtii LSCPPGPELLALLDDFCGRVAELPAEAGLSGQGRKAVQYVRS 113
Volvox corteri LKYKPSQDVTHGLAEFAARVGE---LPRLSSAGRKGVAYVRS 111
Dictyostelium fasciculatum ALHPPSLQILLDLDTFCLGITS------SSILLQKSITYIRA 115
Polysphondylium pallidum SLHPPSLQILHDLDLFCVGISP-----SANATLQKSISYIRA 116
Conserved Region 2
Physcomitrella patens 389 AKR-KNQPSPRWDGETAAGTARRRARP---LEQYRYYSEQQPLKLSEAEMEEVVSAVC 442
Arabidopsis thaliana 357 SKRTRAGPQQIWDDSTVN-TFRPRARP---LEQYRHYSEQQPLRLNPAEVGEVIAAVC 410
Chlamydomonas reinhardtii 440 AARGGGRAGGPGGGGGAGGLGARRGRPARLLEHHPPYNRSEQLPMSEADVAVVVEAVC 497
Volvox corteri 353 AAHGGGGAGGVGRDGAGGG---RRSRPPRLLEHHPPYNRSEPLLMGEADVAVVVEAVC 407
Dictyostelium fasciculatum 427 AKKKAEQ-----KDLTLQFTLRRRKAPA--LEQYKYYSEQEPLKIKDTEKQQVLKVIL 477
Polysphondylium pallidum 484 TKK----------------------------------KDQEPLKISESEKQQVIKVIN 507
Conserved Region 3
Physcomitrella patens 470 DVAASVLIKLLIDMYMADSRAAA-PLTLSLLQGMLSSPQAAVRVRAFDLALNLGVHAHLL 528
Arabidopsis thaliana 437 DVAVSVLIKLVIDMYVLDARIAA-PLTLSMLEEMLCSTKAPCRIRVFDLILNLGVHAQLL 495
Chlamydomonas reinhardtii 553 QMCGSILMKLIIDMWQRCGPVGSYTLVLRMLRAALRSSQPSTRARAFDVLYNLSVHGAML 612
Volvox corteri 464 QICGGILMKLLVDMWLRSEPQEFFIVVLRMLCRALRSTQPSTRARAFDVLYNLYIHGAML 523
Dictyostelium fasciculatum 486 SIATKIFIKIISDIYCKNGVDGE-KIIFGYLKQILESPNRDTRIHLFNIIFNLSVHVNIY 544
Polysphondylium pallidum 516 SIATKIFIKILLDIYCKHGVEGE-KLIQSYFNSIIASPYRDARSHLFNIIFNLSIHINIY 574
Conserved Region 4
Physcomitrella patens 593 VFEAWLLDIVCEMLLYLVQAEETEEGVWSAALSCFLYLVCDRGRICRKRLAGLDIRVLSSLLEVSWMHAWADEVHCRL
Arabidopsis thaliana 544 NFESWILKILFEILLLLVQVEEKEECVWASALSCLLYFICDRGKIRRNQLNGLDIRVIKALLGTSKRNSWSEVVHSKL
Chlamydomonas reinhardtii 821 VYDRWLRALLFQLLCDLCERAEYAEEVWRAAMGATSQLCAHGGHWVAGRVAHLPPAALAGLLRAACNLAWSQELYGHL
Volvox corteri 742 GYDRWLRALLFRLLFELRQMEETSGEVWRAAMGCLAQMCSHGGYWVTAWVQHLPPSALSCLLRVACNFNWSLELYAQL
Dictyostelium fasciculatum 559 ELQDSVFSLLRDCLNQMTQVGEKDNDVWAEALNCVLVFIVEQGNVIRHRLLQLNSQIIGCFLKFIQDS--SDQTKRML
Polysphondylium pallidum 589 DLQESVFSLLREVLLY-CVKHEQDEKVWLEALTCIIFFVVDQGVVIRSRLHSLNSQIIAALLKYANDT--SDQTKRML
Physcomitrella patens IRMACNLLYRQ 681
Arabidopsis thaliana ICIMTNMFYQS 632
Chlamydomonas reinhardtii LRLVPHVLVPP 909
Volvox corteri LKLVPCVLARP 830
Dictyostelium fasciculatum VRMLCNFLYKE 645
Polysphondylium pallidum IRMLCNFLYKD 674
Conserved Region 5
Physcomitrella patens 698 DQLYLMGGVEVICNEYARARTAESKQNLFAVLLDFV 733
Arabidopsis thaliana 651 DQVDLIGGVEYIFFEYSLATTREERRNLYSVLFDYV 686
Chlamydomonas reinhardtii 977 ARLAAFGGLEELLFHEMRAPTPDSRAALLAALAYCC 1012
Volvox corteri 899 RRLGEFGGMQELLFHEVRAPTPDSRASLMVALVQCC 934
Dictyostelium fasciculatum 661 EELNKIGGIDFILNLYTSIRSNEAKNNLFVVIFDYV 696
Polysphondylium pallidum 701 EELNNIGGIDFILRLYTTIRSNEAKNNLFVIIFDYV 736
Conserved Region 6
Physcomitrella patens 1172 DVRAALLLLLIGKCSTYQAALEEVGGEEFFRGLLDDSDARIAYYTSAELLKRMMREEPEAYQHMLHNLVFKAQQSNNE
Arabidopsis thaliana 1087 DIRATLLLLLIGKCTADTVAFQEVGGEEFFRELLDDTDSRVAYYSSAELLKRMMTEEPEKYQNMLQKLVFKAQQSNNE
Chlamydomonas reinhardtii 1732 DRRLALLLLLMARCSLDPEAFAKYSLSHVIKAQLSCEDLRCRYYAGVYLLKHWMLNQHDRYWRSLRHIIGTAQQLNDE
Volvox corteri 1577 DRRLALLLLLIARCSMDPEAFAKYSLSHVVKAQLGCDDLRCRYYAGVYLLKHWMLNQHDKFWRTLRHIIGTAQQLNDE
Dictyostelium fasciculatum 984 EQRLVLVHLLILRCH-DPEDLARVGGIGFFKSLLNDSCTQIAYHSSYELLTQLESESPEQYRSILTRLLSKARENNDE
Polysphondylium pallidum 906 ETRMVLLHLLILRCN-ESEDLAKIGGISFFKNLMNDPCTPIAYHSSYELLTQLEAESPEQYRSILQRLLSKARENNDE
Physcomitrella patens KLLENPYLQMHGILQLSSEMGNTL 1273
Arabidopsis thaliana KLLENPYLQMCGILQLSNEL 1184
Chlamydomonas reinhardtii RLLDNPYLQMRTMLNVDHA 1828
Volvox corteri RLLDNPYLQMRTMLNVDHV 1673
Dictyostelium fasciculatum NLISNPFFQVQGIIEMTHKAS 1081
Polysphondylium pallidum NLISNPFFQVQGIIDMTHKT 1002
