# Supplementary material for: Conditional genetic screen in Physcomitrella patens reveals a novel microtubule depolymerizing-end-tracking protein
Source: PLoS Genet. 2018 May 10;14(5):e1007221. doi: 10.1371/journal.pgen.1007221 (PMC5944918; doi:10.1371/journal.pgen.1007221)
Supplement: S1 Table — (PDF) [file pgen.1007221.s008.pdf]

Supplemental Table S1. Number of CLoG1 genes present in different fully-sequenced plant and algal genomes.

Species	Number of genes
Physcomitrella patens	1
Selaginella moellendorffii	1
Brachypodium distachyon	1
Zea mays	1
Setaria italica	1
Panicum virgatum	2
Aquilegia coerulea	1
Eutrema salsugineum	1
Boechera stricta	1
Capsella grandiflora	1
Capsella rubella	1
Arabidopsis lyrata	1
Arabidopsis thaliana	1
Eucalyptus grandis	1
Vitis vinifera	1
Mimulus guttatus	1
Cucumis stativus	1
Fragaria vesca	1
Malus domestica	1
Prunus persica	1
Glycine max	4
Medicago truncatula	1
Phaseolus vulgaris	1
Citrus clementina	1
Citrus sinensis	1
Gossypium raimondii	2
Theobroma cacao	1
Linum usitatissimum	1
Manihot esculenta	1
Ricinus communis	1
Populus trichocarpa	2
Salix purpurea	2
Coccomyxa subellipsoidea	1
Chlamydomonas reinhardtii	1

Genome sequences were obtained from *Phytozome*. Note that with the exception of a few species most plants have only one copy of the CLoG1 gene.
