# Supplementary material for: Conditional genetic screen in Physcomitrella patens reveals a novel microtubule depolymerizing-end-tracking protein
Source: PLoS Genet. 2018 May 10;14(5):e1007221. doi: 10.1371/journal.pgen.1007221 (PMC5944918; doi:10.1371/journal.pgen.1007221)
Supplement: S2 Table — (PDF) [file pgen.1007221.s009.pdf]

Supplemental Table S2. Approximate chromosome lengths of *P. patens* used in the Monte Carlo simulation.

Chromosome	Chromosome lengths (bp)
1	30,000,000
2	25,000,000
3	25,000,000
4	22,000,000
5	20,000,000
6	19,000,000
7	18,000,000
8	17,000,000
9	17,000,000
10	17,000,000
11	17,000,000
12	17,000,000
13	17,000,000
14	17,000,000
15	16,000,000
16	16,000,000
17	15,000,000
18	15,000,000
19	15,000,000
20	15,000,000
21	15,000,000
22	15,000,000
23	15,000,000
24	13,000,000
25	11,000,000
26	10,000,000
27	5,000,000

The chromosome lengths were obtained from an unpublished V3 *P. patens* genome assembly (*Physcomitrella patens v3.0 early release*).
