# Supplementary material for: Conditional genetic screen in Physcomitrella patens reveals a novel microtubule depolymerizing-end-tracking protein
Source: PLoS Genet. 2018 May 10;14(5):e1007221. doi: 10.1371/journal.pgen.1007221 (PMC5944918; doi:10.1371/journal.pgen.1007221)
Supplement: S3 Table — (PDF) [file pgen.1007221.s010.pdf]

Supplemental Table S3. Approximate chances of 0, 1, and 2 crossovers(s) on 27 chromosomes of *P. patens*.

Chromosome	Chances of 0, 1 and 2 crossovers (from left to right)		
1	0.10	0.30	0.60
2	0.15	0.38	0.47
3	0.15	0.38	0.47
4	0.18	0.43	0.39
5	0.20	0.47	0.33
6	21	0.48	0.31
7	0.22	0.50	0.28
8	0.23	0.52	0.25
9	0.23	0.52	0.25
10	0.23	0.52	0.25
11	0.23	0.52	0.25
12	0.23	0.52	0.25
13	0.23	0.52	0.25
14	0.23	0.52	0.25
15	0.24	0.53	0.23
16	0.24	0.53	0.23
17	0.25	0.55	0.20
18	0.25	0.55	0.20
19	0.25	0.55	0.20
20	0.25	0.55	0.20
21	0.25	0.55	0.20
22	0.25	0.55	0.20
23	0.25	0.55	0.20
24	0.27	0.58	0.15
25	0.29	0.62	0.09
26	0.30	0.63	0.07
27	0.10	0.30	0.60

The chances of 0, 1, and 2 crossover(s) on each chromosome of *P. patens* were calculated according to the approximate relationship between chromosome lengths and chances of crossovers in outcrossed F2 *Arabidopsis* determined by linear regression (Salomé et al., 2012).

Chance of 0 crossover = -0.01*chromosome length (Mbp)+0.04;

Chance of 1 crossover = -1/60*chromosome length+0.8.
