# Supplementary material for: Conditional genetic screen in Physcomitrella patens reveals a novel microtubule depolymerizing-end-tracking protein
Source: PLoS Genet. 2018 May 10;14(5):e1007221. doi: 10.1371/journal.pgen.1007221 (PMC5944918; doi:10.1371/journal.pgen.1007221)
Supplement: S4 Table — (PDF) [file pgen.1007221.s011.pdf]

Supplemental Table S4. Scale and shape parameters of the gamma distribution which determines the distance between the first and second crossovers in the Monte Carlo simulation.

Chromosome	Scale and shape parameters of gamma distribution	
1	2506000	4.9229
2	2255000	4.5279
3	2255000	4.5279
4	2104400	4.3779
5	2004000	4.2779
6	1953800	4.2279
7	1903600	4.1779
8	1853400	4.1279
9	1853400	4.1279
10	1853400	4.1279
11	1853400	4.1279
12	1853400	4.1279
13	1853400	4.1279
14	1853400	4.1279
15	1803200	4.0779
16	1803200	4.0779
17	1753000	4.0279
18	1753000	4.0279
19	1753000	4.0279
20	1753000	4.0279
21	1753000	4.0279
22	1753000	4.0279
23	1753000	4.0279
24	1652600	3.9279
25	1552200	3.8279
26	1502000	3.7779
27	1251000	3.5279

The scale and shape parameters of the gamma distribution of each *P. patens* chromosome were calculated separately according to their approximate relationship with chromosome lengths determined by linear regression in outcrossed F2 *Arabidopsis* (Salomé et al., 2012).

Scale parameter = 0.0526*chromosome length (bp)+10^6 (R2=0.8835);

Shape parameter = 5*10^{-8}*chromosome length (bp)+3.2779 (R2=0.7476).
