# Supplementary material for: Conditional genetic screen in Physcomitrella patens reveals a novel microtubule depolymerizing-end-tracking protein
Source: PLoS Genet. 2018 May 10;14(5):e1007221. doi: 10.1371/journal.pgen.1007221 (PMC5944918; doi:10.1371/journal.pgen.1007221)
Supplement: S5 Table — (PDF) [file pgen.1007221.s012.pdf]

Supplemental Table S5. Primers used in this study

Primer Name	Primer Sequence	Notes
CLoG1-mut(F)	ATTCCTCCTCCTCTACTTCCAACT	Amplify CLoG1 locus
CLoG1-mut(R)	GCGTTGAAGTCATCTGTAATGAGG	Amplify CLoG1 locus
CLoG1-outF	TACCTGAAA AACTCTTCACCACCA	Amplify CLoG1 locus-sequence verify
CLoG1-outR	GTTCACTATTGGAGGTAAGCTGGA	Amplify CLoG1 locus-sequence verify
CLoG1-inF	TTTGCTTTCAATAAACCGCACAAC	Sequencing of PCR product
CLoG1-inR	TGTTGTGTTGTTCTTTGAGCAGTT	Sequencing of PCR product
mutCLoG1-2F	GTTCTTCCAAGCCTGCACGACC	Amplify-sequence different mutant locus
mutCLoG1-2R	TGCTTTAAGAACTCCATTTCCAAG	Amplify-sequence different mutant locus
CLoG1 full cds F	CACCATGTCGTTAGCAGAGCAGGA	CLoG1 cDNA clonning
CLoG1 full cds R	TCA AAGAGTGTTCCCCATTTCACT T	CLoG1 cDNA clonning
CLoG1UTRi500bpF	CACCGGAGTTGCGAGGTTTGGGAC	CLoG1-RNAi construct
CLoG1UTRiR	CCTGCCAGTTTCTAAAGAGA	CLoG1-RNAi construct
CLoG1 FL seq 1F	TGAGAGAGGTCGCAGCCCACCTGT	CLoG1-cDNA sequencing
CLoG1 FL seq 2F	ATGTGCTAAAATGGCGGTGGGCAG	CLoG1-cDNA sequencing
CLoG1 FL seq 3F	AGCACTGCTTCTACTCCGGCCCAT	CLoG1-cDNA sequencing
CLoG1 FL seq 4F	GGAGGGAGTGTGGTCTGCTGCATT	CLoG1-cDNA sequencing
CLoG1 FL seq 1R	GTTGGCGGAACTTGCAAGCTGCAT	CLoG1-cDNA sequencing
CLoG1 FL seq 2R	TCTCTATTTCCAATCCTGGTCTCT	CLoG1-cDNA sequencing
CLoG1 FL seq 3R	GCATCTAATGATGCCGTGATGTCA	CLoG1-cDNA sequencing
attB1CLoG1F	GGGGACAAGTTTGTACAAAAAAGCAGGC TTAATGTCGTTAGCAGAGCAGGAATC	Multisite Gateway Clonning of CLoG1
attB5rCLoGR	GGGGACAACTTTTGTATACAAAGTTGTAA GAGTGTTCCCCATTTCACTTG	Multisite Gateway Clonning of CLoG1
attB5CLoG1F	GGGGACAACTTTGTATACAAAAGTTGTGT CGTTAGCAGAGCAGGAATCCTC	Multisite Gateway Clonning of CLoG1
attB2CLoG1R	GGGGACCACTTTGTACAAGAAAGCTGGG TATCAAAGAGTGTTCCCCATTTCAC	Multisite Gateway Cloning of CLoG1
