# Supplementary material for: Conditional genetic screen in Physcomitrella patens reveals a novel microtubule depolymerizing-end-tracking protein
Source: PLoS Genet. 2018 May 10;14(5):e1007221. doi: 10.1371/journal.pgen.1007221 (PMC5944918; doi:10.1371/journal.pgen.1007221)
Supplement: S6 Table — (PDF) [file pgen.1007221.s013.pdf]

Supplemental Table S6. Species, accession numbers, and amino acid sequence lengths of CLoG1 protein homologues used in this study.

Species Name	Accesion number	Sequence Length
Aquilegia coerulea	Aquca_010_00719.1	1213
Arabidopsis lyrata	AL3G24260.t1	1182
Arabidopsis thaliana	AT3G12590.1	1184
Boechera stricta	Bostr.3640s0032.1.p	1184
Brachypodium distachyon	Bradi2g62330.1.p	1249
Capsella grandiflora	Cagra.5641s0023.1.p	1180
Capsella rubella	Carubv10015495m	1180
Chlamydomonas reinhardtii	Cre17.g707100.t1.1	1828
Citrus clementina	Ciclev10030542m	1202
Citrus sinensis	orange1.1g000952m	1210
Cucumis sativus	Cucsa.165310.1	1210
Dictyostelium discoideum	DDB_G0278301	1148
Dictyostelium fasciculatum	DFA1505604	1081
Eucalyptus grandis	Eucgr.G02920.1	1196
Eutrema salsugineum	Thhalv10019927m	1185
Fragaria vesca	mrna26647.1-v1.0-hybrid	1256
Glycine max-1	Glyma.20G099500.1.p	1206
Glycine max-2	Glyma.10G289700.1.p	1206
Glycine max-3	Glyma.02G093600.1.p	1207
Glycine max-4	Glyma.18G288500.1.p	1199
Gossypium raimondii-1	Gorai.012G069700.1	1213
Gossypium raimondii-2	Gorai.011G255800.1	1219
Linum usitatissimum	Lus10005160	1222
Manihot esculenta	Manes.10G033000.1	1215
Marchantia polymorpha	Mapoly0126s0012.1	1336
Medicago truncatula	Medtr7g024190.1	1193
Mimulus guttatus	Migut.F01518.1.p	1196
Panicum virgatum-1	Pavir.Ea04151.1.p	1209
Panicum virgatum-2	Pavir.Eb04061.1.p	1214
Phaseolus vulgaris	Phvul.008G013500.2	1195
Physcomitrella patens	Pp3c24_6470V3.1 (MG754010)	1273
Polysphondylium pallidum	PPA1363332	1002
Populus trichocarpa-1	Potri.008G052700.1	1186
Populus trichocarpa-2	Potri.010G207900.1	1221
Prunus persica	ppa000390m	1215
Ricinus communis	29585.m000590	1206
Salix purpurea-1	SapurV1A.0038s0880.1.p	1153
Salix purpurea-2	SapurV1A.0095s0460.1.p	1170
Selaginella moellendorffii	413864	1086
Setaria italica	Si000093m	1200
Sphagnum fallax	Sphfalx0136s0011.1	1296
Theobroma cacao	Thecc1EG042817t3	1218
Vitis vinifera	GSVIVT01016585001	1188
Volvox carteri	Vocar.0021s0063.1	1673
Zea mays	GRMZM2G459642_P01	1121
